# Insulin signaling and glucose metabolism in different hepatoma cell lines deviate from hepatocyte physiology toward a convergent aberrant phenotype

**DOI:** 10.1038/s41598-020-68721-9

**Published:** 2020-07-21

**Authors:** Angela Molinaro, Barbara Becattini, Giovanni Solinas

**Affiliations:** The Wallenberg Laboratory and Sahlgrenska Center for Cardiovascular and Metabolic Research, Department of Molecular and Clinical Medicine, Institute of Medicine, University of Gothenburg, Gothenburg, Sweden

**Keywords:** Cancer, Cell biology, Physiology, Diseases, Endocrinology, Gastroenterology, Medical research, Molecular medicine, Oncology, Pathogenesis

## Abstract

Hepatoma cell lines are widely used to model the hepatocyte for insulin signaling and fatty liver disease. However, a direct comparison of insulin action in primary hepatocytes and in hepatoma cell lines is needed to validate this model and to better understand liver cancer. Here we have investigated insulin signaling, gluconeogenic gene expression, glucose production, and fatty acid synthase abundance in primary hepatocytes and in HepG2, Hepa 1–6, and McARH7777 hepatoma cell lines. Differences in the electrophoretic profiles of protein extracts from human and mouse primary hepatocytes and the hepatoma cells lines are shown. Compared to primary hepatocytes, hepatoma cells showed high basal phosphorylation of AKT at Thr 308 and constitutively activated RAS-MAPK signaling, which were resistant to the dominant negative Ras mutant H-Ras17N. Hepatoma cell lines also showed defective expression of gluconeogenic enzymes, insulin unresponsive GSK phosphorylation, and marginal glucose production. Hepatoma cells also showed lower protein levels of fatty acid synthase and a largely distinct protein electrophoresis profile from hepatocytes but similar between different hepatoma lines. We conclude that hepatoma cell lines do not accurately model the hepatocyte for insulin action but may be valuable tools to investigate the proteomic changes conferring to hepatocellular carcinoma its peculiar metabolisms.

## Introduction

Obesity and type-2 diabetes have reached unprecedented proportions and may be the largest pandemic in the history of humanity^1^. Furthermore, fatty liver disease is a condition closely associated with obesity and insulin resistance, which at advanced stage progresses to cirrhosis and hepatocellular carcinoma, and it was predicted that fatty liver disease will be the first cause for liver transplantation in the near future^2^. The liver is a major insulin target organ, is the main source of endogenous glucose production, plays a chief role in the control of systemic lipid metabolism, and is central to the link between obesity and type-2 diabetes^3–5^. Hence, identifying the molecular mechanisms linking obesity to the pathogenesis of hepatic insulin resistance and the progression of fatty liver disease is a major challenge of modern biomedical research.

The metabolic function of the liver is highly integrated with other organs and insulin action on the hepatocyte implicates indirect mechanisms involving signals from adipocytes and the brain^4,6^. However, recent studies indicate that direct insulin action on the hepatocyte plays a dominant role in the control of glucose metabolism^7,8^. A better understanding of the direct insulin action on hepatocyte metabolism in physiological conditions and in obesity is therefore necessary to unravel the link between obesity, insulin resistance, and fatty liver disease. This research field to progress needs a solid cell culture model to investigate and define the molecular mechanisms of insulin action in the hepatocyte and its role in metabolic homeostasis and disease progression. Cultures of hepatoma-derived cell lines display typical morphological features of hepatocytes, express specific hepatocyte markers and therefore can be seen as a practical and ethical alternative to primary hepatocyte cell cultures. Indeed, preparation of primary hepatocytes requires animals and is more technically demanding and labor intensive than immortalized cell lines. Furthermore, the availability of hepatoma cell lines of human origin, such as HepG2, could also be considered a significant advantage, as human primary hepatocytes have a limited availability at a prohibitive cost. It is therefore not surprising that thousands of studies used hepatoma cell lines, most commonly HepG2 cells, to model hepatocytes in insulin signaling or in metabolism. However, whereas HepG2 proteome was shown to be qualitatively similar to the one of human primary hepatocytes^9^, principal component analysis of these proteomes could distinguish between these cell-types, indicating significant quantitative differences^9,10^. Most importantly, insulin action in hepatoma cell lines remains largely uncharacterized. To our knowledge, only one recent study has directly compared insulin action in HepG2 and other immortalized hepatocyte cell lines, to the one in primary mouse hepatocytes, and several metabolic differences between these cell types were found^11^. However, the authors could not measure insulin-induced phosphorylation of insulin-receptor and AKT in HepG2 cells, probably because of technical difficulties, and have not evaluated MAPK signaling, which complicates the interpretation of this study. Furthermore, another recent study compared HepG2 cells with two lines of immortalized hepatocytes for insulin action and hepatokine gene expression^12^. This study concluded that HepG2 cells are a valid model to investigate insulin signaling, and that HepG2 show a gluconeogenic and hepatokine gene-expression pattern similar to the one observed in in-vivo settings^12^. Yet, this study has not directly compared HepG2 and immortalized hepatocytes to primary cell cultures, or to the liver in in-vivo settings.

Overall, a direct comparison of insulin signaling in primary hepatocytes and in hepatoma cell lines is necessary to validate hepatoma cell lines as model to study insulin signaling and insulin action on glucose metabolism, and to better understand the biology of hepatocellular carcinoma (HCC).

Here we have investigated insulin-driven PI3K and RAS-MAPK signaling, gluconeogenic gene expression, glucose production, and fatty acid synthase (FAS) protein levels in primary mouse hepatocytes and in three hepatoma cell lines from three different species: human HepG2; mouse Hepa 1–6 cells; and rat McARH7777. Finally, a Coomassie gel of protein extracts from primary human and mouse hepatocytes and from the three hepatoma cell lines above is shown to estimate the relative differences in protein expression profiles between species and between primary hepatocytes and hepatoma cells.

## Results

### Human HepG2 hepatoma cells display aberrantly activated RAS-MAPK and altered PI3K-AKT signaling

**Figure 1:**
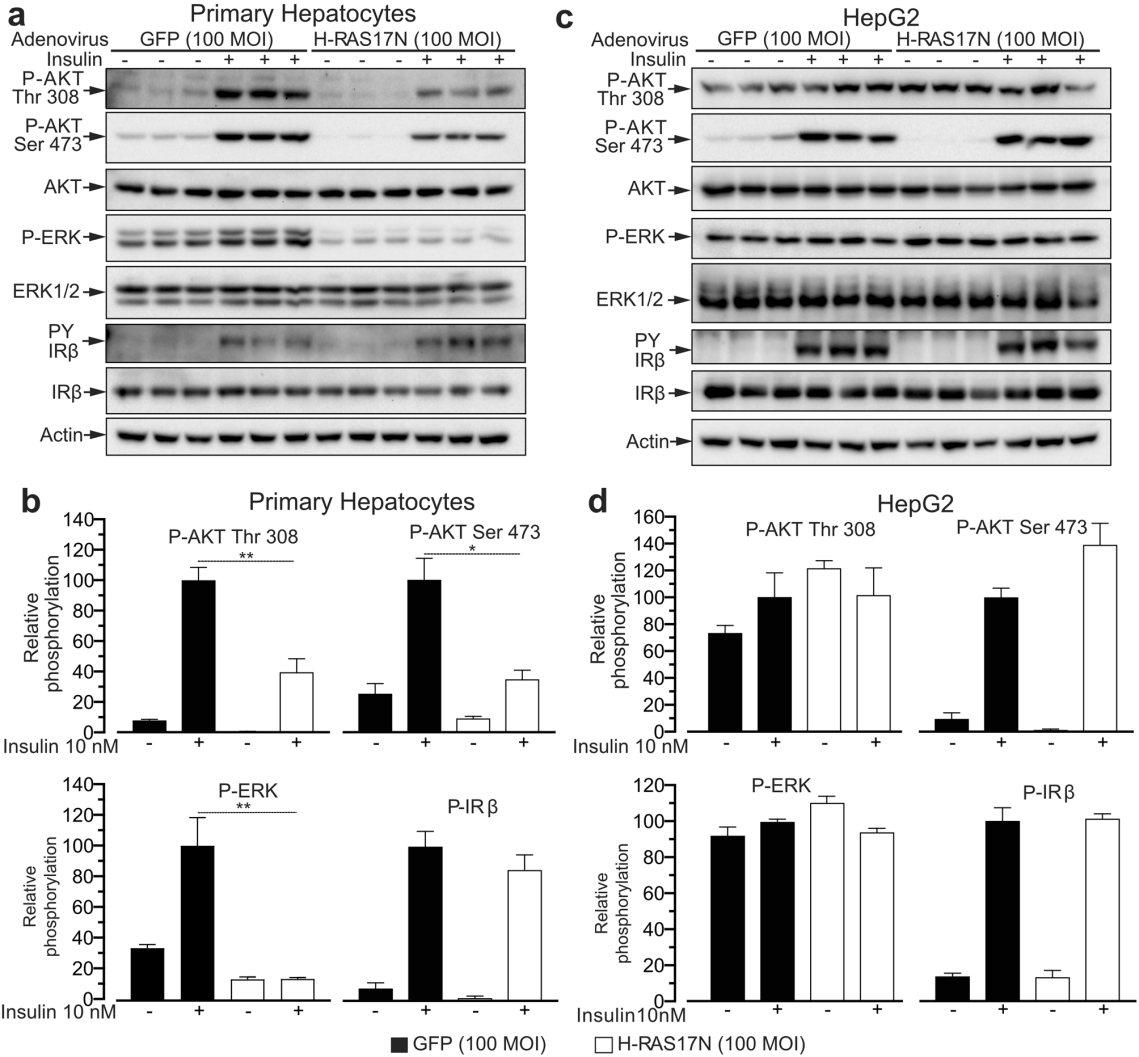
The HepG2 human hepatoma cell line displays aberrant insulin signaling compared to murine primary hepatocytes. (**a**) Immunoblot analysis of insulin signaling in primary mouse hepatocytes infected with a control adenovirus expressing the GFP or a virus expressing the negative dominant RAS mutant H-RAS17N and stimulated with 10 nM insulin for 8 min. (**b**) Quantification of blots in (**a**). (**c**) Immunoblot analysis of insulin signaling and insulin receptor phosphorylation performed as in (**a**) but using the HepG2 cell line. (**d**) Quantification of blots in (**c**). n = 3 biological replicates (mice) for primary hepatocytes and 3 independent experiments for HepG2. Data are represented as mean ± SEM.

The dominant negative RAS mutant H-RAS17N completely blunts insulin driven MAPK signaling and partially reduces insulin-driven AKT phosphorylation in primary mouse and human hepatocytes^7^. To evaluate insulin signaling in HepG2 hepatoma cells we have measured insulin-induced receptor beta chain (IRβ) autophosphorylation, AKT phosphorylation, and ERK phosphorylation in HepG2 cells in parallel to primary mouse hepatocytes infected either with a control virus expressing the GFP or with a virus expressing the dominant negative mutant H-RAS17N (Fig. 1).

Both primary hepatocytes and HepG2 cells showed insulin-induced IRβ phosphorylation which was not affected by the H-RAS17N mutant. However, whereas in primary hepatocytes ERK phosphorylation was significantly induced by insulin and blunted by the H-RAS17N mutant (Fig. 1a, b), HepG2 cells showed constitutively elevated basal levels of ERK phosphorylation which were insensitive to the effects of insulin or of H-RAS17N (Fig. 1c,d). A similar pattern was observed for AKT Thr 308 phosphorylation, which was strongly induced by insulin and partially reduced by H-RAS17N in primary hepatocytes (Fig. 1a, b), while HepG2 cells showed high basal AKT Thr 308 phosphorylation which was not significantly elevated by insulin or reduced by H-RAS17N (Fig. 1c, d). Basal AKT Ser 473 phosphorylation was low in both unstimulated primary hepatocytes and HepG2 and robustly induced by insulin in both cell types, yet it was significantly reduced by H-RAS17N in primary hepatocytes but not in HepG2 cells.

Altogether, compared to primary hepatocytes, HepG2 hepatoma cells displayed elevated basal ERK and AKT Thr 308 phosphorylation which were not significantly elevated by insulin and that were insensitive to the effects of the dominant negative mutant H-RAS17N. We observed a normal insulin induction of AKT Ser 473 phosphorylation in HepG2 cells that, differently from primary hepatocytes, was resistant to the effects of H-RAS17N. Because insulin receptor autophosphorylation was similar in primary hepatocytes and in HepG2, our data indicate that HepG2 cells display post-insulin receptor aberrant RAS-MAPK signaling and altered PI3K-AKT signaling.

### Mouse Hepa 1–6 and rat McARH7777 hepatoma cells also display aberrantly activated RAS-MAPK and altered PI3K-AKT signaling

**Figure 2:**
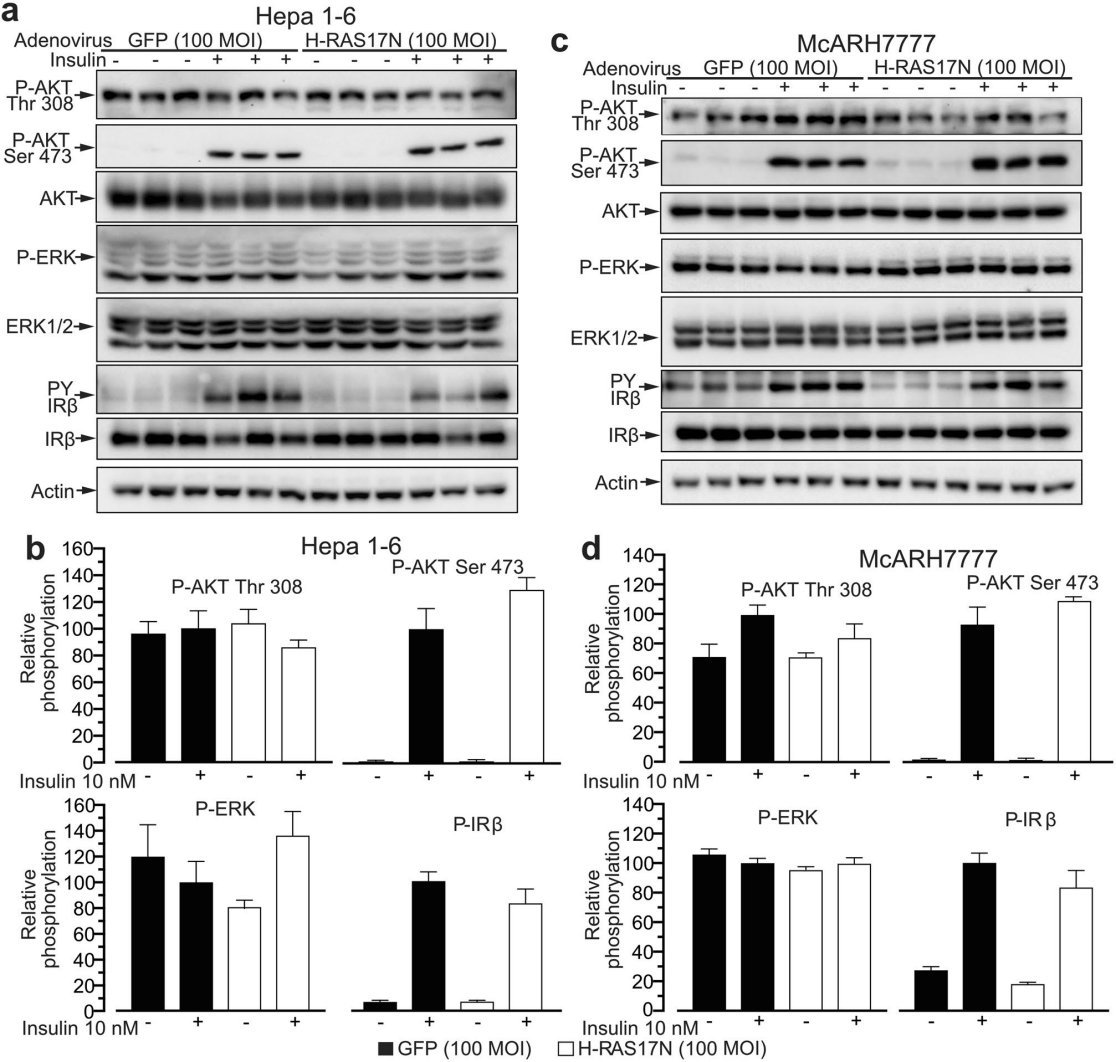
Murine hepatoma cell line Hepa 1–6 and rat hepatoma cell line McARH7777 display aberrant insulin signaling. (**a**) Immunoblot analysis of insulin signaling in the Hepa 1–6 cells infected with 100 MOI of a control adenovirus expressing the GFP or a virus expressing the negative dominant RAS mutant H-RAS17N and stimulated with 10 nM insulin for 8 min. (**b**) Quantification of blots in (**a**). (**c**) Immunoblot analysis of insulin signaling and insulin receptor phosphorylation as in (**a**) was performed using the McARH7777 cell line. (**d**) Quantification of blots in (**c**). n = 3 independent experiments. Data are represented as mean ± SEM.

To investigate whether the derangement in insulin signaling observed in human HepG2 cells is common to other hepatoma cell lines we performed a similar experiment to the one described above in Fig. 1 but using mouse Hepa1-6 and rat McARH7777 hepatoma cells (Fig. 2).

For both Hepa1-6 and McARH7777 we observed the same derangement we found in HepG2: normal insulin-induced IRβ phosphorylation, but constructively elevated basal levels of ERK and elevated basal AKT Thr 308 phosphorylation, which were not significantly increased by insulin or inhibited by H-RAS17N. However, these hepatoma cell lines showed little or no basal AKT Ser 473 phosphorylation which was potently induced by insulin but that was not affected by H-RAS17N (Fig. 2). Measurement of infection efficiency showed that 50 MOI of GFP adenovirus were sufficient to infect 100% of the three hepatoma cell lines we have investigated (Supplementary Fig. S1), which is similar to what we have found in primary hepatocytes^7^. Furthermore, 100 MOI of H-RAS17N adenovirus overexpressed the H-RAS17N transgene to a similar extent in primary hepatocytes, HepG2, McARH7777, and Hepa1-6 (Supplementary Fig. S2). Finally, insulin dose response analysis showed that the three hepatoma cell lines displayed an overall normal insulin-sensitivity for IRβ and AKT Ser 473 phosphorylation (Supplementary Fig. S3). Altogether these results indicate that, similarly to human HepG2 hepatoma cells, McARH7777 rat and Hepa1-6 mouse hepatoma cells display aberrant post receptor insulin signaling consisting in elevated basal phosphorylation of ERK and AKT Thr 308, and insensitivity to the effects of dominant negative H-RAS17N mutant.

### Hepatoma cells display blunted and insulin irresponsive glucose production

The fact that we observed a similar perturbation of insulin signaling in different hepatoma cell lines derived from independent clones from different animal species motivated us to investigate the action of insulin on its gluconeogenic targets and on glucose production.

**Figure 3:**
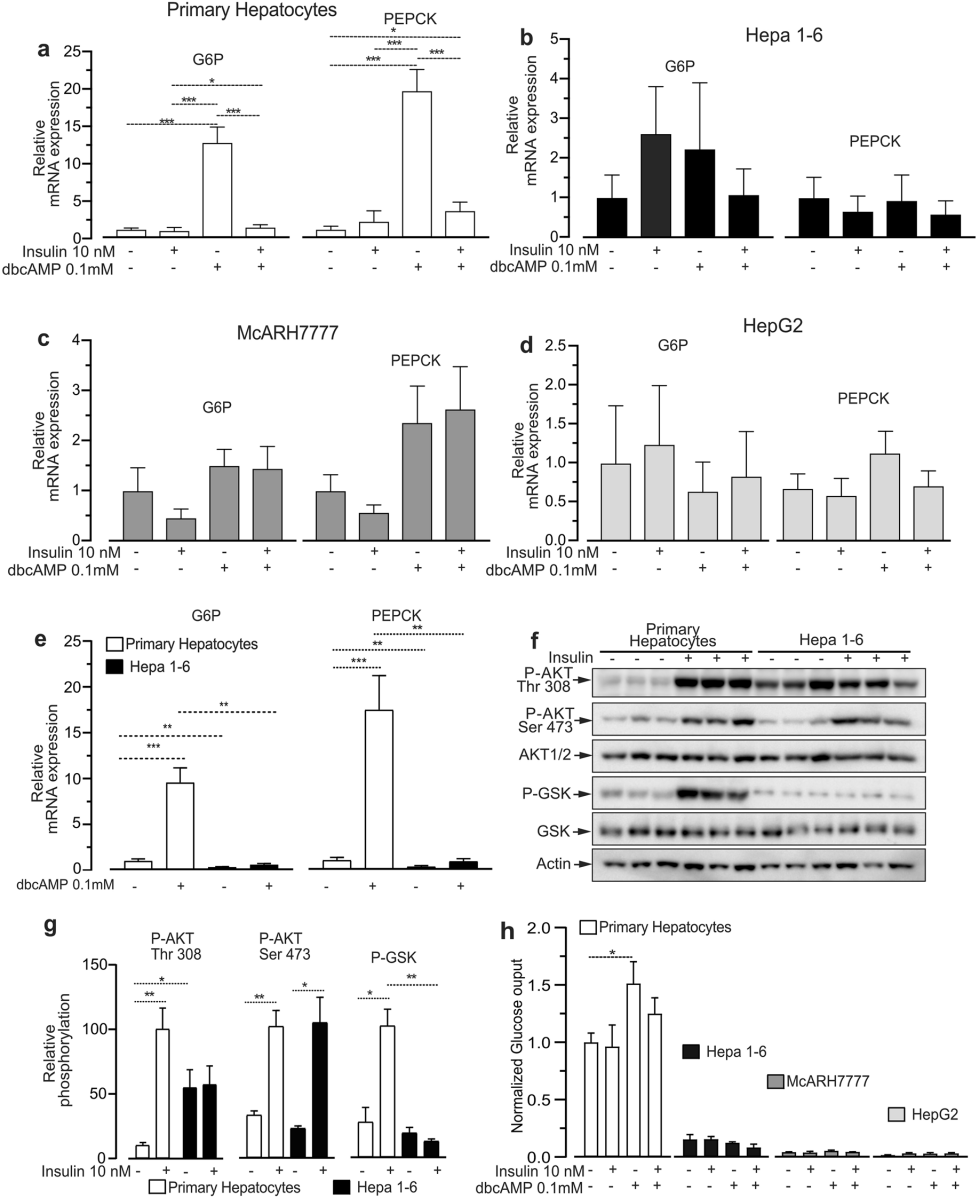
Hepatoma cell lines display severely impaired gluconeogenic gene expression and blunted glucose production. (**a**) qPCR analysis of mRNA levels of glucose 6 phosphatase (G6P) and phosphoenolpyruvate carboxykinase (PEPCK) in primary mouse hepatocytes exposed for 6 h to 100 µM of dbcAMP, or 10 nM insulin, or 100 µM dbcAMP and 10 nM insulin. (**b**) qPCR analysis of mRNA levels of G6P and PEPCK in Hepa 1–6 cells treated as in (**a**). (**c**) qPCR analysis of mRNA levels of G6P and PEPCK in McARH7777 cell line treated as in (**a**). (**s**) qPCR analysis of mRNA levels of G6P and PEPCK in HepG2 cells treated as in (**a**). (**e**) Direct comparison of basal and cAMP-induced G6P and PEPCK mRNA levels in primary mouse hepatocytes and mouse Hepa 1–6. (**f**) Immunoblot analysis of insulin-driven AKT Thr 308 phosphorylation, AKT Ser 473 phosphorylation and GSK3β phosphorylation in primary hepatocytes and Hepa 1–6 cells stimulated for 8 min with 10 nM insulin. (**g**) Quantification of blots in (**f**). (**h**) Relative glucose production in primary hepatocytes, Hepa 1–6, McARH7777, and HepG2 stimulated with 100 µM dbcAMP in presence or not of 10 nM insulin for 6 h. n = 8 for (**a**); n = 5 for (**b**); n = 8 for (**c**); n = 7 for (**d**); and (**e**) n = 8 for heaptocytes and n = 5 for Hepa 1–6 n = 3 for (**f**, **g**) n = 3; for (**h**) n = 5 hepatocytes and n = 4 for each hepatoma cell line. n indicates biological replicates for primary hepatocytes, and independent experiments for cell lines. Data are represented as mean ± SEM.

Primary hepatocytes, HepG2, Hepa1-6, and McARH7777 hepatoma cell lines were treated either with the cell permeable cAMP analog dbcAMP, to induce gluconeogenic gene expression, or with insulin, or dbcAMP in presence of insulin. In primary hepatocytes dbcAMP caused a strong induction of the key gluconeogenic genes glucose 6 phosphatase (G6P) and phosphoenolpyruvate carboxykinase (PEPCK), which was dampened to basal levels by insulin (Fig. 3a). However, we could not observe any significant effect of either dbcAMP or insulin on both G6P or PEPCK gene expression in all the tested hepatoma cell lines (Fig. 3b–d). Since primary hepatocytes and Hepa1-6 are both of mouse origin we could directly compare PEPCK and G6P mRNA levels in these cells. Our data indicate that, compared to primary hepatocytes Hepa1-6 hepatoma cells display lower basal PEPCK and G6P mRNA levels, and virtually absent induction by dbcAMP (Fig. 3e).

We next evaluated the effects of insulin on glycogen synthase kinase phosphorylation (GSK), a major molecular effector of the insulin-PI3K-AKT signaling cascade mediating insulin action on glucose mobilization from glycogen. The results indicate that despite the fact that insulin induced AKT Ser 473 phosphorylation to a similar extent in primary hepatocytes and hepatoma cells GSK phosphorylation was significantly increased by insulin only in primary hepatocytes (Figs. 3f, g, Supplementary Fig. S4a, b).

Finally, to investigate the physiological relevance of these derangements we measured glucose production in primary hepatocytes and in the three hepatoma cell lines exposed to dbcAMP, insulin, or dbcAMP in presence of insulin.

Primary hepatocytes displayed a substantial basal glucose production, which was significantly increased by dbcAMP, but not by dbcAMP in presence of insulin (Fig. 3h). However, compared to primary hepatocytes, HepG2, Hepa1-6, and McARH7777 showed severely blunted or marginal glucose production rates, which were not significantly affected by either dbcAMP or insulin (Fig. 3h).

Overall, HepG2, Hepa1-6, and McARH7777 hepatoma cells display similar derangements of the glucose production molecular machinery, consisting in very low glucose production rates and aberrant expression of gluconeogenic genes which were unresponsive to either dbcAMP or insulin. Finally, the hepatoma cell lines largely dissociated insulin-induced AKT Ser 473 from GSK phosphorylation.

### Protein expression in different hepatoma cell lines drastically deviates from the primary hepatocyte profile and converge toward a similar pattern

**Figure 4:**
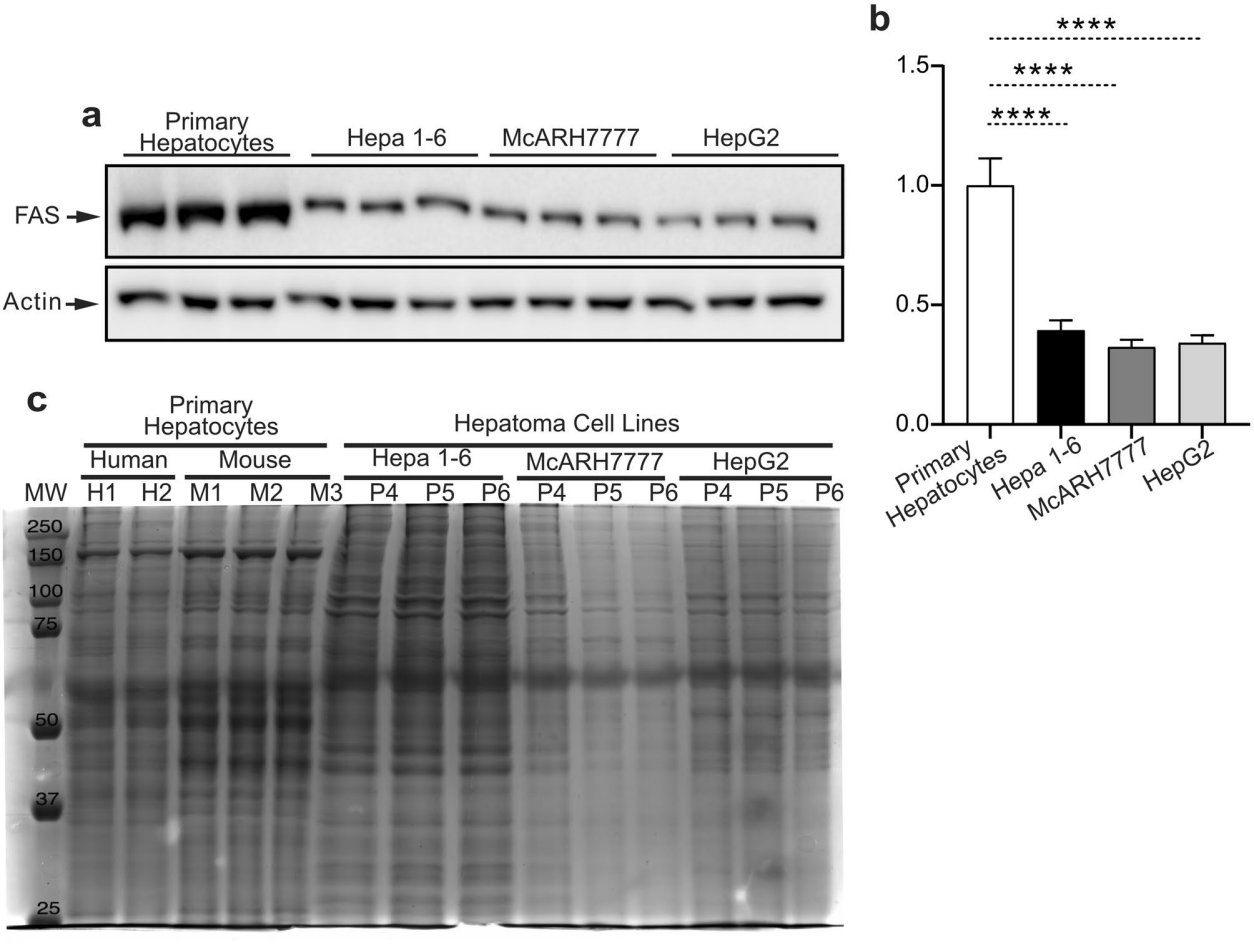
Hepatoma cell lines display reduced fatty acid synthase expression and a distinct protein electrophoresis pattern. (**a**) Immunoblot analysis of FAS on primary hepatocytes, Hepa 1–6, McARH7777, and HepG2. (**b**) Quantification of blots in (**a**). (**c**) Coomassie staining of a protein electrophoresis of extracts from human primary hepatocytes, murine primary hepatocytes, Hepa 1–6, McARH7777, and HepG2. n = 9 for a and b; n = 2–3 for c. n indicates biological replicates for primary hepatocytes, technical replicates for human hepatocytes, and independent experiments for cell lines. Data are represented as mean ± SEM.

Our data from above show that HepG2, Hepa1-6, and McARH7777 hepatoma cells display similarly aberrant insulin signaling and glucose production machinery. We have therefore compared the protein levels of the lipogenic enzyme fatty acid synthase (FAS), in primary hepatocytes with the one of the three hepatoma cell types, in three independent experiments each one with three replicates. The results show that FAS expression was significantly reduced in all the hepatoma cells analyzed (Fig. 4a, b Supplementary Fig. S4c, d).

Overall, the expression of all the metabolic enzymes we have measured was quantitatively different between primary hepatocytes and hepatoma cells but similar between the different hepatoma cell lines that we have tested (Figs. 3a–g, 4a, b). This observation motivated us to estimate the difference in protein expression “landscape” between primary human and mouse hepatocytes and human HepG2, rat Hepa1-6, and mouse McARH7777 hepatoma cells. To achieve this aim we performed a simple Coomassie staining of a gel electrophoresis of protein extracts from either human or mouse primary hepatocytes and the three different hepatoma cell lines. Protein extracts from three independent primary hepatocyte preparations from three different mice showed electrophoretic profiles which were identical between them but that could be distinguished from protein extracts of human primary hepatocytes (Fig. 4c). However, human and mouse primary hepatocytes showed an overall similar protein electrophoresis profile. By contrast, the protein electrophoretic profile of human HepG2, rat Hepa1-6, and mouse McARH7777 hepatoma cells was remarkably distinct from the one of primary hepatocytes but was largely similar among them.

Altogether, our data indicate that quantitative differences in protein expression profile between hepatoma cells and primary hepatocytes appear to be larger than the one between mouse and human hepatocytes, and that the three hepatoma cell lines derived from mouse, rat, and human display a similar protein expression profile.

## Discussion

We have found that, compared to primary hepatocytes, hepatoma cell lines display aberrant insulin—receptor tyrosine kinase signaling consisting in elevated basal phosphorylation of AKT specifically at Thr 308 and constitutively activated RAS-MAPK signaling, which were resistant to the effects of the dominant negative Ras mutant H-RAS17N. Compared to primary hepatocytes, these hepatoma cell lines also showed defective expression of the key gluconeogenic genes G6P and PEPCK, insulin unresponsive GSK, and dampened glucose production which was unresponsive to either dbcAMP or insulin. Hepatoma cell lines also showed lower protein levels of FAS compared to primary hepatocytes, and showed a protein electrophoresis profile which was remarkably distinct from the one of hepatocytes but similar between them despite being derived from different animal species.

These results are relevant to the interpretation of a large bulk of scientific literature where these hepatoma cells have been used to model the hepatocyte to investigate insulin signaling, insulin resistance, glucose and lipid metabolism for the study of diabetes and fatty liver disease. These data are also relevant to the field of hepatocellular carcinoma, as we have found that different hepatoma cell lines derived from different tumor clones from three different animal species and that have been cultured for years, appear to converge toward similar derangements of PI3K-AKT and RAS-MAPK signaling, glucose metabolism, and protein expression profile. It is thus reasonable that this aberrant phenotype could be a general hallmark of HCC, and the full mechanistic elucidation of such phenotype may lead to new pharmacological targets for the treatment of HCC. Indeed, it was shown that the gene expression profile of several hepatoma cell lines is closely correlated with the one of primary tumors, with the HepG2 cell line showing the stronger correlation^13^. Furthermore, it was reported that mice injected with H22 carcinoma cells develop tumors in their liver that showed largely reduced PEPCK and G6P expression compared to normal liver tissue^14^. The latter is consistent with our results and indicate that the blunted glucose production we observed in cultured hepatoma cell lines is also observed in-vivo.

Whereas we have found that FAS levels were significantly reduced in cultured HCC cells compared to primary hepatocytes, it was shown that the expression of FAS and other DNL markers is elevated in primary HCC compared to normal liver of patients^15^. This apparent discrepancy can be explained by the fact that, in the normal hepatocyte, DNL is a highly regulated process which is minimal in animals fed with diets abundant in lipids, but that it can be dramatically induced during refeeding on a diet rich in carbohydrates after a period of starvation^5,16^. Because in our study cells are cultured in medium containing only glucose as energy source (but not lipids), it is expected that DNL is activated in the cultured primary hepatocytes.

Our immunoblots from primary hepatocytes and from hepatoma cell lines were performed with antibodies from the same tube, which in principle should exclude the possibility that the elevated signals we measured for AKT Thr 308 and ERK phosphorylation in hepatoma cells but not in hepatocytes, are caused by an artefactual interaction with non-specific bands. However, it is likely that others before us have observed a similar pattern of AKT Thr 308 and ERK phosphorylation in hepatoma cells and ascribe it to suboptimal antibody quality or immunoblot conditions. Indeed, out of 100 randomly selected manuscripts measuring insulin-induced AKT phosphorylation in the hepatoma cell lines that we have read, only seven studies specifically showed AKT phosphorylation at Thr 308, whereas the other 93 either showed only AKT Ser 473 phosphorylation or a non-specified AKT phosphorylation site. Mechanistically, the constitutively activated ERK signaling and elevated AKT Thr 308 basal phosphorylation observed in the hepatoma cells are expected to be caused by genetic mutations. Thereby, we have examined the information available on the HepG2 cell line at Broad Institute Cancer Cell Line encyclopedia (CCLE), where we have found 386 entries for mutations in the HepG2 genome. These included the well-established NRAS activating mutation Q61L, which fully explains the constitutively elevated ERK phosphorylation and the insensitivity to the effects of the dominant negative H-Ras17N mutant that we observed in HepG2 hepatoma cells. Furthermore, according to data from CCLE, HepG2 also bear a S265R missense mutation on MAPK4, a kinase which was shown to directly phosphorylate AKT specifically on Thr 308^17^.

Other mutations reported at CCLE for the HepG2 genome, which are expected to affect PI3K or MAPK signaling are a G573S substitution on IGF2R, and a nonsense mutation on the PI3K regulatory subunit PIK3R2, whereas a D69N mutation for FOXO1 is expected to affect gluconeogenic gene-expression. Although these mutations may contribute to the blunted glucose production observed in hepatoma cells, this dampened glucose production may be more generally linked to the ensemble of genetic aberrations driving altered glucose metabolism in HCC. Furthermore, the genetic and epigenetic alterations driving aberrant metabolism in HCC most likely involve indirect and complex interactions, which are not readily deducible from genetic and epigenetic signatures. However, our data indicate that hepatoma cell lines bear a conserved proteomic signature whose quantitative comparison to the one of primary hepatocytes may reveal the detailed mechanism causing the “malignant” metabolic transformation of hepatocellular carcinoma.

Altogether, our data indicate that hepatoma cell lines from different species largely deviate from the original hepatocyte phenotype for insulin signaling, insulin action on glucose production, and protein expression signature.

This observation implies that the large bulk of literature using hepatoma cell lines, such as HepG2, to model the hepatocyte for insulin action on glucose and lipid metabolism, should be carefully interpreted in light of the data in this manuscript and from emerging evidence from other studies^11^. However, it is worth of attention that three different hepatoma cell lines from three different species, which have been cultured for years, show remarkable similarities in their aberrant phenotype for insulin signaling, glucose production, and protein electrophoretic profile. Thereby we conclude that hepatoma cell lines are most likely valuable tools to investigate the proteomic changes conferring to HCC its peculiar metabolisms.

## Methods

### Mice

Mice used for this study were wild type C57BL/6J. Mice were maintained at the EBM specific pathogen free facility of the University of Gothenburg at room temperature of 22 °C under 12 h of light and 12 h of dark cycles. All the protocols were carried out according to the relevant guidelines and regulations and were authorized by the Ethics Committee on Animal Care Use in Gothenburg, Sweden.

### Primary hepatocytes and hepatoma cell lines

Murine primary hepatocytes were prepared from 12–18 weeks old mice. Mice were anesthetized with isofluorane (Baxter KDG9623) and sacrificed by bleeding. We perfused the liver with 60 ml of washing solution 1 × Hank`s buffered salt sodium [GIBCO 14170-112] in absence of Magnesium and Calcium with addition of 0.5 mM EGTA. After washing, the liver was perfused with a digestion medium: DMEM-low glucose (HyClone) with 1% Penicillin Streptomycin and 15 mM HEPES and 0.8 mg/ml of collagenase type IV (SIGMA). The digestion process was performed at 37 °C for 8–10 min, when liver structure is disrupted, Glisson’s capsule was opened releasing the hepatocytes in a 10 cm Petri dish filled with 10 ml digestion medium. Cells are filtered on a 70 µM cell strainer and collected by centrifugation (3 min at 5×*g*) and washed three times with 20–25 ml of culturing medium: 50% Dulbecco’s Modified Eagle’s Medium (DMEM) high-glucose, 50% HAM’S F-12, 10% FBS, 1% Penicillin Streptomycin and 100 µM dexamethasone. Alive cells were counted by trypan blue exclusion and plated 400,000 cells/ml in 12 well plates pre-coated one day earlier with type I collagen dissolved in 0.02 N acetic acid let dry overnight. From a 12–18 weeks old mouse we typically obtained 30–50 million hepatocytes with 80–98% viability.

The hepatoma cell lines were kindly provided by Prof. Stefano Romeo (all the cell lines) and by Prof. Fredrik Bäckhed (an independent stock of Hepa 1–6), which were purchased from ATCC and all experiments were performed between passage 4 and 6. HepG2 cells (from a human hepatoma) were cultured at 37 °C, 5% CO_2_ in Eagle’s Minimum Essential Medium (EMEM) with 10% FBS, 1% Penicillin Streptomycin. Hepa 1–6 cells (from mouse hepatoma) were cultured in Dulbecco’s Modified Eagle’s Medium (DMEM) high glucose with 10% FBS and 1% Penicillin Streptomycin. McARH7777 cells (from rat hepatoma) were cultured in Dulbecco’s Modified Eagle’s Medium (DMEM) high glucose with 10% FBS and 1% Penicillin Streptomycin.

Human hepatocyte lysate was purchased from Sciencell.

### Viral vectors and cell cultures treatments

The procedures to generate, amplify, and titer the viral vectors used in this manuscript were previously described^7^. Brifely, the empty VmAdcDNA3 virusmid used to generate the recombinant adenoviral vectors and the control adenovirus expressing the green fluorescent protein were kindly gifted by Luciano Pirola at INSERM 1,060 CarMeN Lyon. The recombinant adenovirus expressing the H-RAS17N dominant negative mutant was generated by homologous recombination of the VmAdcDNA3 virusmid and a pcDNA3.1 plasmid in which was cloned the H-RAS17N cDNA fused with three HA tags at the N terminus. The pcDNA3.1 H-RAS17N 3XHA plasmid was bought from the UMR cDNA Resource Center, University of Missouri-Rolla (GB Acc. NM_005343). For immunoblot analysis cells were serum starved for 12 h and stimulated with 10 nM of insulin or with the indicated dose (for dose response analysis) for 8 min. When indicated cells were infected 24 h before insulin treatment with either 100 MOI of either a GFP-expressing control adenovirus, or an adenovirus expressing the dominant-negative mutant H-RAS17N.

To estimate the infection efficiency, cells were infected with 25, 50 and 100 MOI of the GFP adenovirus and 24 h post-infection the number of GFP positive cells was evaluated. Primary hepatocytes and hepatoma cell lines were also infected with 100 MOI of H-RAS17N and relative protein levels were compared by immunoblot. For qPCR analysis of phosphoenolpyruvate carboxykinase (PEPCK) and glucose 6-phosphate (G6P) gene expression, and measurement of glucose production, cells were serum starved for 12 h and then treated either with 10 nM of insulin, 100 μM of dbcAMP, or 10 nM insulin and 100 μM dbcAMP for 6 h.

### Real time qPCR analysis of gene expression

The RNA was extracted from primary hepatocytes and cell lines with guanidinium-thiocyanate method. The complementary DNA was prepared using reverse transcription kit (Promega A3802). qPCR was performed using SYBR green mix (Bio-Rad 172-5274) and the following primers: mouse G6P fwd 5′-CGACTCGCTATCTCCAAGTGA-3′, rev 5′GTTGAACCAGTCTCCGACCA3′; mouse PEPCK fwd 5′-AGCATTCAACGCCAGGTTC-3′, PEPCK rev 5′-CGAGTCTGTCAGTTCAATACCAA-3′; rat G6P fwd 5′-GGCTCACTTTCCCCA-3′, rev 5′-ATCCAAGTGCGAAACCAAACA-3′; rat PEPCK fwd 5′-GGTTGCAGGCCCAGTTGTTG-3′, rev 5′-GAC AGACTCGCCCTATGTGGTG-3′; human G6P fwd 5′-GCTTCGCCATCGGATTTTAT-3′, rev 5′-CACCACCTCTGGGCTTTCT-3′; human PEPCK fwd 5′-GCAAGATTATCGTCACCC-3′ and rev 5′-GGCATTGAACGCTTTCTCAAAAT-3’.

### Immunoblots and Coomassie staining

For immunoblot, proteins were separated by SDS-PAGE electrophoresis and transferred to a PVDF membrane. Membranes were incubated for 1 h in blocking solution 3% bovine serum albumin (BSA) 0.3% tween in phosphate saline buffer; and then incubated with the primary antibody overnight at 4 °C in PBS 3% BSA 0.3% tween. Membranes were washed 3 times in PBS with 0.3% tween and incubated with horseradish peroxidase conjugated secondary antibody (GE Healthcare) in PBS 0.3% tween 3% BSA for one hour at room temperature. After three washes in PBS 0.3% tween, membranes were incubated with detection reagent and the signal was analyzed through BioRad ChemiDoc apparatus and BioRad Image Lab software.

All primary antibodies were from Cell Signaling except for anti-phospho tyrosine 4G10 (Millipore), anti-FAS (abcam), and anti-H-RAS (Santa Cruz).

For coomassie staining, the acrylamide gel after the electrophoresis was covered with a warm fixation solution (50% EtOH, 7% Acetic Acid) for about one minute, and then incubated in 50 ml of 5% EtOH, 7.5% acetic acid to which were freshly added 0.5 ml of Coomassie stock solution (50% EtOH, 10% acetic acid 0.25% Coomassie blue B-250) and warmed to about 50–60 °C in a microwave for about 1 min. The gel was then incubated for 10–20 min at room temperature on a shaker, and then destained with destained solution (5% EtOH, 7.5% Acetic Acid) for 3 h at room temperature on a shaker. Image acquisition was performed with BioRad ChemiDoc apparatus and BioRad Image Lab software Version 6.0.0 build 26 2017, Bio-Rad Laboratories (https://www.bio-rad.com/en-se/product/image-lab-software?ID=KRE6P5E8Z).

### Measurement of glucose production

Glucose production in cultured cells was measured using a commercially available hexokinase-based glucose production assay kit (Abcam). After 12 h starvation cells were treated with 10 nM of insulin, or with 100 µM dbcAMP, or with both 10 nM insulin and 100 μM dbcAMP added in the cell medium for 6 h. 50 μl of medium was collected and mixed with an equal volume of reaction mix. A standard curve was prepared by serial dilutions of a 1.25 mM NADH stock in assay buffer in as indicated by the vendor, and 450 nm absorbance was measured 30 min and 60 min after plating.

### Statistical analysis

Data are displayed as means and the error bands represent standard errors. For “biological replicate” is intended a primary hepatocyte preparation derived from one mouse. Values are expressed as relative to controls. P-values for immunoblot signals on the same gel, and for glucose production analysis were performed by two-tailed t-test, while Mann–Whitney test was applied to qPCR analysis of gene expression and for analysis of immunoblots data from different membranes (in the three independent FAS measurements). All the statistical analysis was performed with the GraphPad Prism software.

## Data availability

Unprocessed blot images are provided in the supplemental material of this manuscript, and datasets from this study are available from the corresponding author on reasonable request.

## Supplementary information

Supplementary figures S1–S4.

Supplementary information.

## Abbreviations

Ras: Retrovirus-associated DNA sequences
MAPK: Mitogen activated protein kinase
PI3K: Phosphoinositide 3 kinase
GSK: Glycogen synthase kinase
G6P: Glucose 6 phosphatase
PEPCK: Phosphoenolpyruvate carboxykinase
FAS: Fatty acid synthase
Ser: Serine
Thr: Threonine
