# Supplementary material for: Insulin signaling and glucose metabolism in different hepatoma cell lines deviate from hepatocyte physiology toward a convergent aberrant phenotype
Source: Sci Rep. 2020 Jul 21;10:12031. doi: 10.1038/s41598-020-68721-9 (PMC7374613; doi:10.1038/s41598-020-68721-9)
Supplement: Supplementary file 1 — Supplementary figures S1–S4. [file 41598_2020_68721_MOESM1_ESM.pdf]

Insulin Signaling and Glucose Metabolism in Different Hepatoma Cell Lines Deviate from Hepatocyte Physiology Toward a Convergent Aberrant Phenotype

Angela Molinaro[1], Barbara Becattini[1], Giovanni Solinas[1*]

1) The Wallenberg Laboratory and Sahlgrenska Center for Cardiovascular and Metabolic Research, Department of Molecular and Clinical Medicine, Institute of Medicine, University of Gothenburg, Gothenburg, Sweden.

* Corresponding author. E-mail: Giovanni.Solinas@wlab.gu.se

Supplementary Figures S1-S4.

Supplementary Figure 1

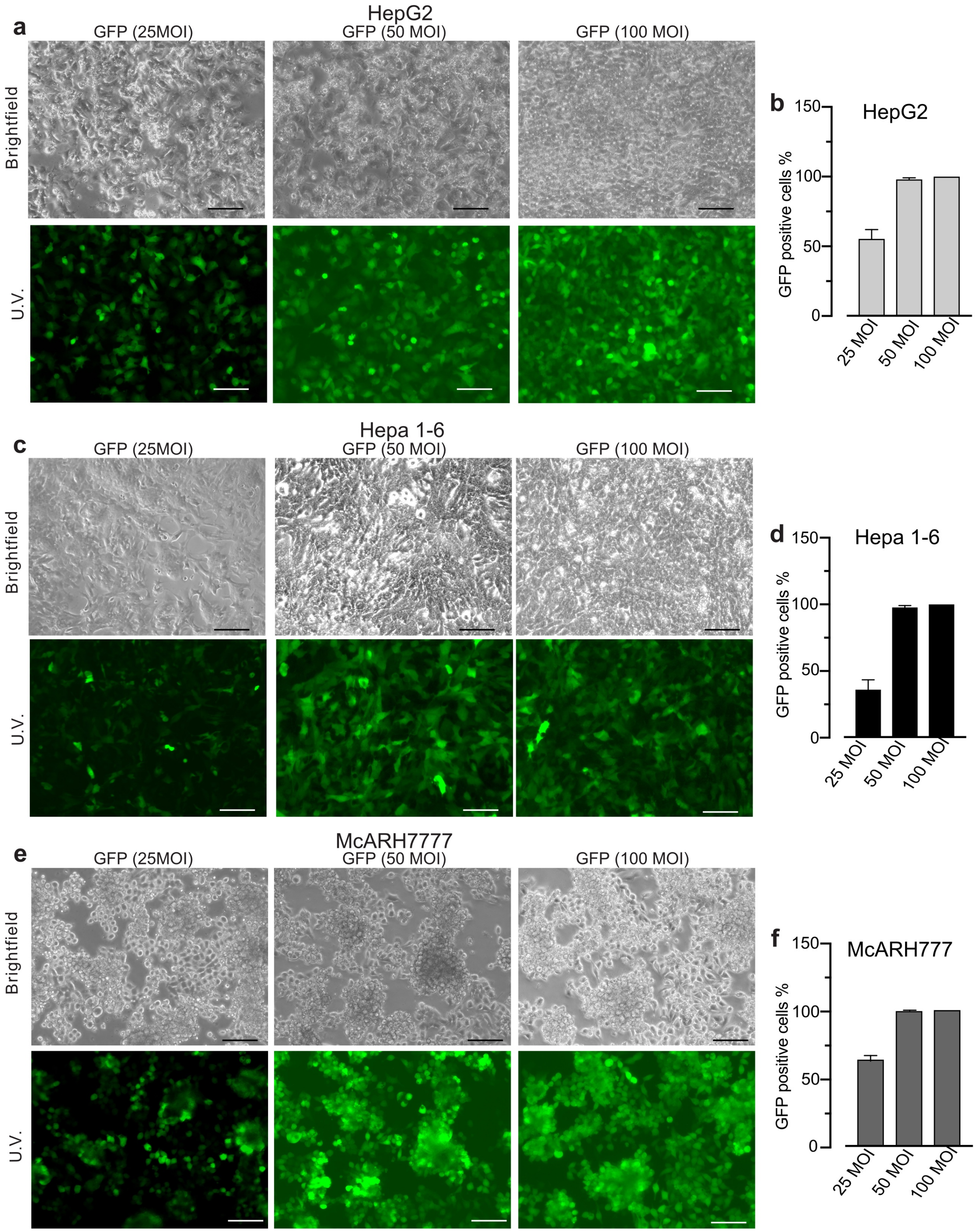

Supplementary Figure 1. Adenoviruses efficiency of infection. (**a**) Brightfield and U.V. fluorescence images of HepG2 infected with different MOI of a recombinant adenovirus expressing the GFP protein. (**b**) Quantification of GFP positive cells in (a). (**c**) Brightfield and U.V. fluorescence images of mouse Hepa 1-6 infected with different MOI of a recombinant adenovirus expressing the GFP protein. (**d**) Quantification of GFP positive cells in (c). (**e**) Brightfield and U.V. fluorescence images of mouse McARH7777 infected with different MOI of a recombinant adenovirus expressing the GFP protein. (**f**) Quantification of GFP positive cells in (e). n=3 independent experiments. Data are represented as mean ± SEM.

Supplementary Figure 2

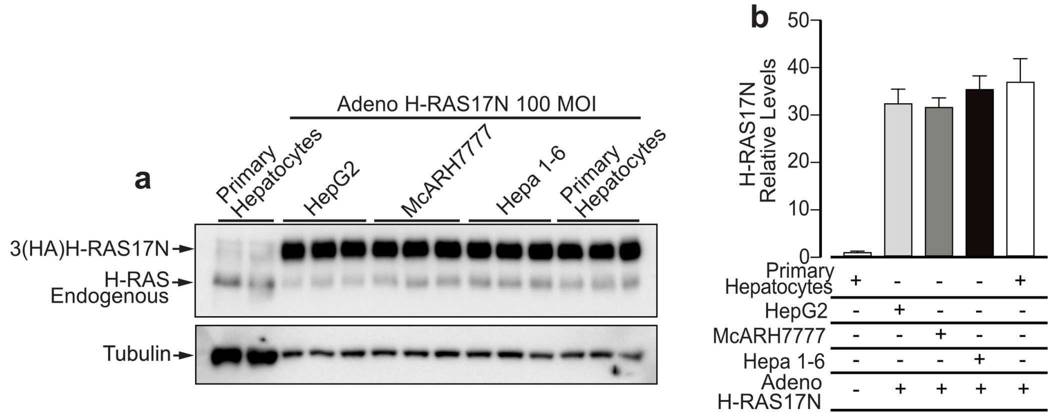

Supplementary Figure 2. Expression of HRAS17N in different hepatoma cell lines. (**a**) Immunoblot analysis of HRAS protein levels in primary hepatocytes not infected, and in HepG2, McARH7777, Hepa 1-6 and primary hepatocytes infected with 100 MOI of an adenovirus expressing the dominant negative RAS mutant H-RAS17N. (**b**) Quantification of (a).

n=2-3 biological replicates. n indicates biological replicates for primary hepatocytes, and independent experiments for cell lines. Data are represented as mean ± SEM.

Supplementary Figure 3

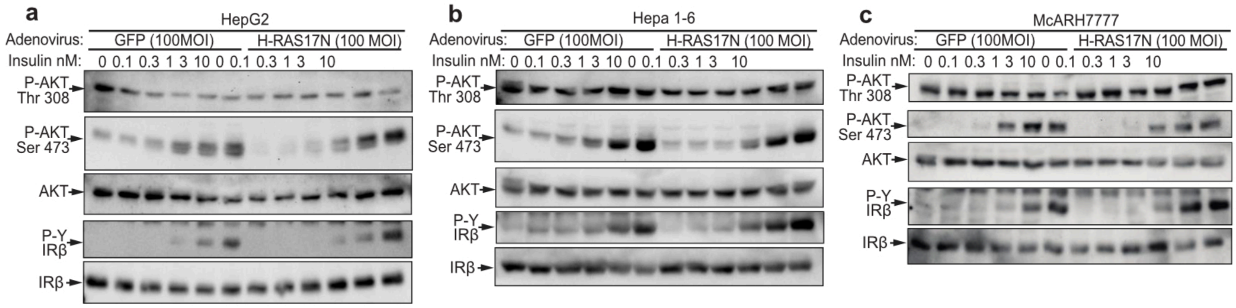

Supplementary Figure 3. Dose Response in the hepatoma cell lines. Immunoblot analysis of an insulin dose response for AKT and insulin receptor phosphorylation in (**a**) HepG2; (**b**) Hepa 1-6; and (**c**) McARH7777 hepatoma cell lines. Cells were stimulated for 8 min with increasing doses of insulin as indicated. For each cell lines 2-3 independent experiments were performed and representative blots are shown.

Supplementary Figure 4

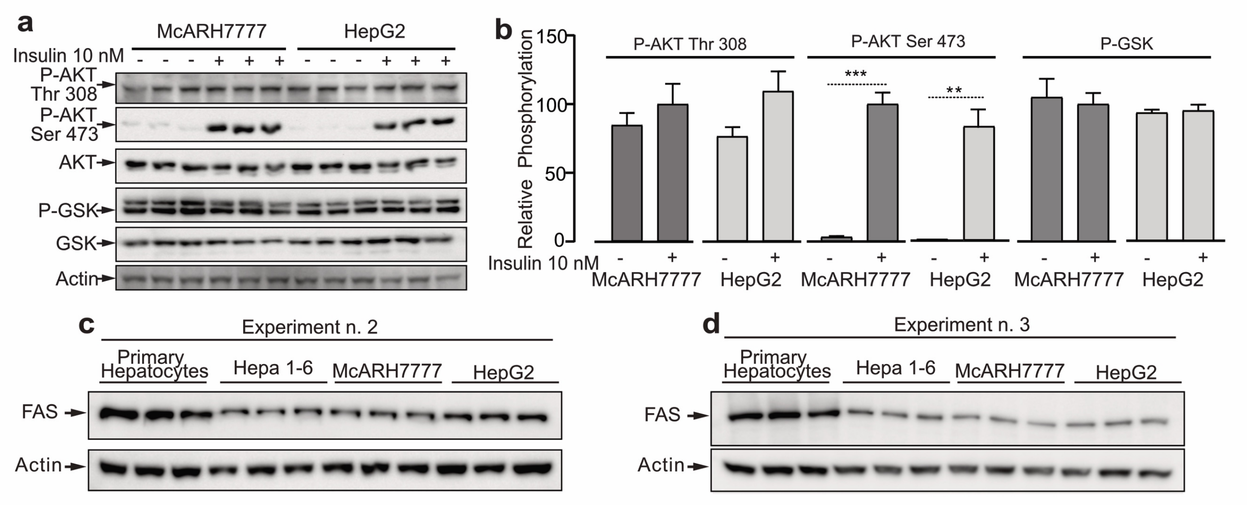

Supplementary Figure 4. McARH7777 and HepG2 display insulin unresponsive GSK phosphorylation. (**a**) Immunoblot analysis of insulin-driven Akt Thr 308 phosphorylation, Akt Ser 473 phosphorylation, and GSK3β phosphorylation in McARH7777 and HepG2 stimulated for 8 min with 10 nM insulin. (**b**) Quantification of blots in A. (**c**) Immunoblot analysis of FAS levels in primary hepatocytes, Hepa 1-6, McARH7777, and HepG2 (experiment n.2 related to Figure 4a, b). (**d**) Immunoblot analysis of FAS of experiment n.3 on primary hepatocytes, Hepa 1-6, McARH7777, and HepG2 (experiment n.3 related to Figure 4a, b). n=3 replicates per experiments.
