# Supplementary material for: Insulin signaling and glucose metabolism in different hepatoma cell lines deviate from hepatocyte physiology toward a convergent aberrant phenotype
Source: Sci Rep. 2020 Jul 21;10:12031. doi: 10.1038/s41598-020-68721-9 (PMC7374613; doi:10.1038/s41598-020-68721-9)
Supplement: Supplementary file 2 — Supplementary information. [file 41598_2020_68721_MOESM2_ESM.pdf]

Insulin Signaling and Glucose Metabolism in Different Hepatoma Cell Lines Deviate from Hepatocyte Physiology Toward a Convergent Aberrant Phenotype

Angela Molinaro[1], Barbara Becattini[1], Giovanni Solinas[1*]

1) The Wallenberg Laboratory and Sahlgrenska Center for Cardiovascular and Metabolic Research, Department of Molecular and Clinical Medicine, Institute of Medicine, University of Gothenburg, Gothenburg, Sweden.

* Corresponding author. E-mail: Giovanni.Solinas@wlab.gu.se

Supplementary materials: unprocessed full blots

Figure 1A

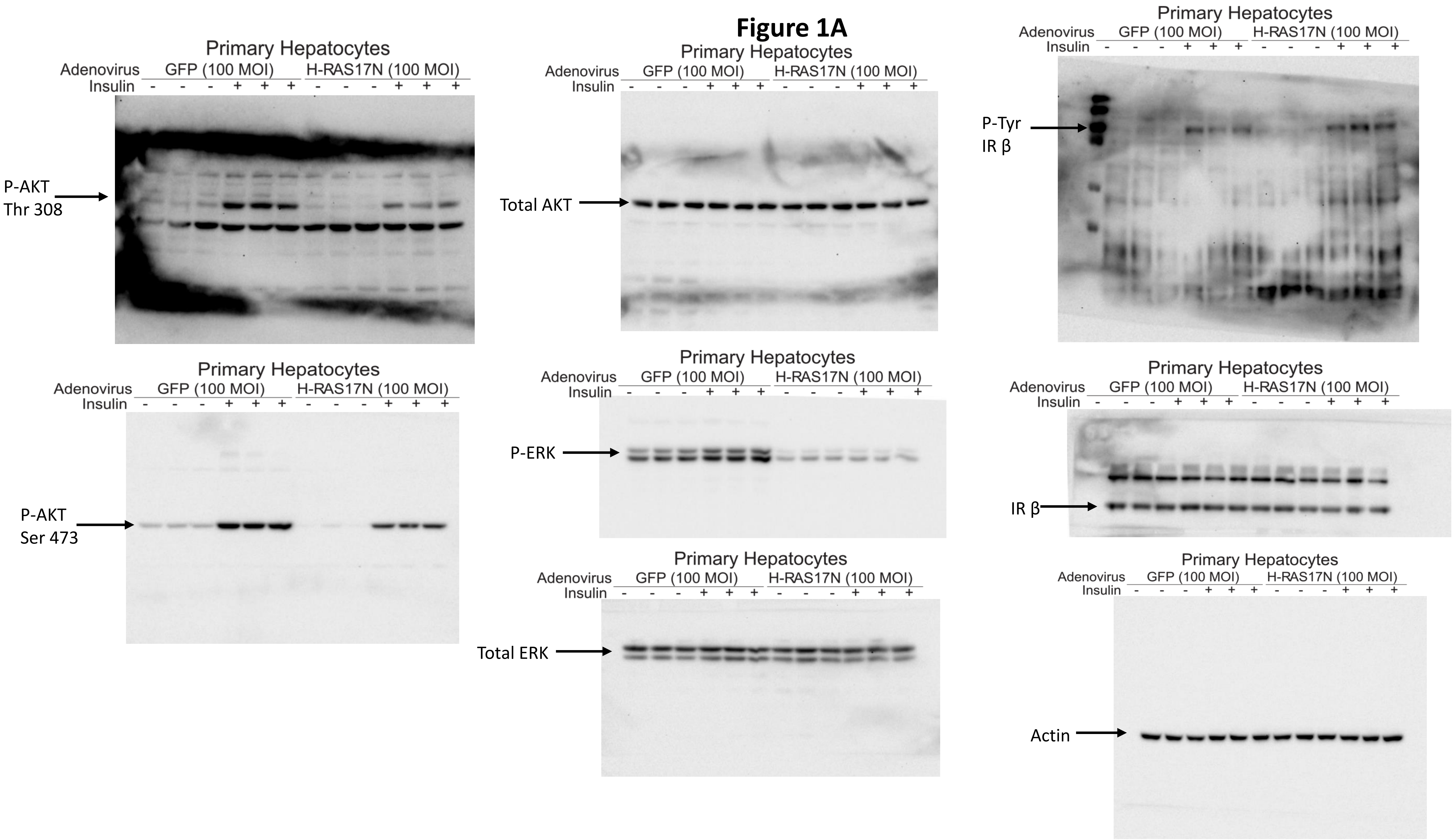
Primary Hepatocytes
Adenovirus GFP (100 MOI) H-RAS17N (100 MOI)
Insulin - - - + + + - - - + + +
P-AKT Thr 308
Primary Hepatocytes
Adenovirus GFP (100 MOI) H-RAS17N (100 MOI)
Insulin - - - + + + - - - + + +
Total AKT
Primary Hepatocytes
Adenovirus GFP (100 MOI) H-RAS17N (100 MOI)
Insulin - - - + + + - - - + + +
P-Tyr IR β
Primary Hepatocytes
Adenovirus GFP (100 MOI) H-RAS17N (100 MOI)
Insulin - - - + + + - - - + + +
P-AKT Ser 473
Primary Hepatocytes
Adenovirus GFP (100 MOI) H-RAS17N (100 MOI)
Insulin - - - + + + - - - + + +
P-ERK
Primary Hepatocytes
Adenovirus GFP (100 MOI) H-RAS17N (100 MOI)
Insulin - - - + + + - - - + + +
IR β
Primary Hepatocytes
Adenovirus GFP (100 MOI) H-RAS17N (100 MOI)
Insulin - - - + + + - - - + + +
Total ERK
Primary Hepatocytes
Adenovirus GFP (100 MOI) H-RAS17N (100 MOI)
Insulin - - - + + + - - - + + +
Actin

Figure 1C

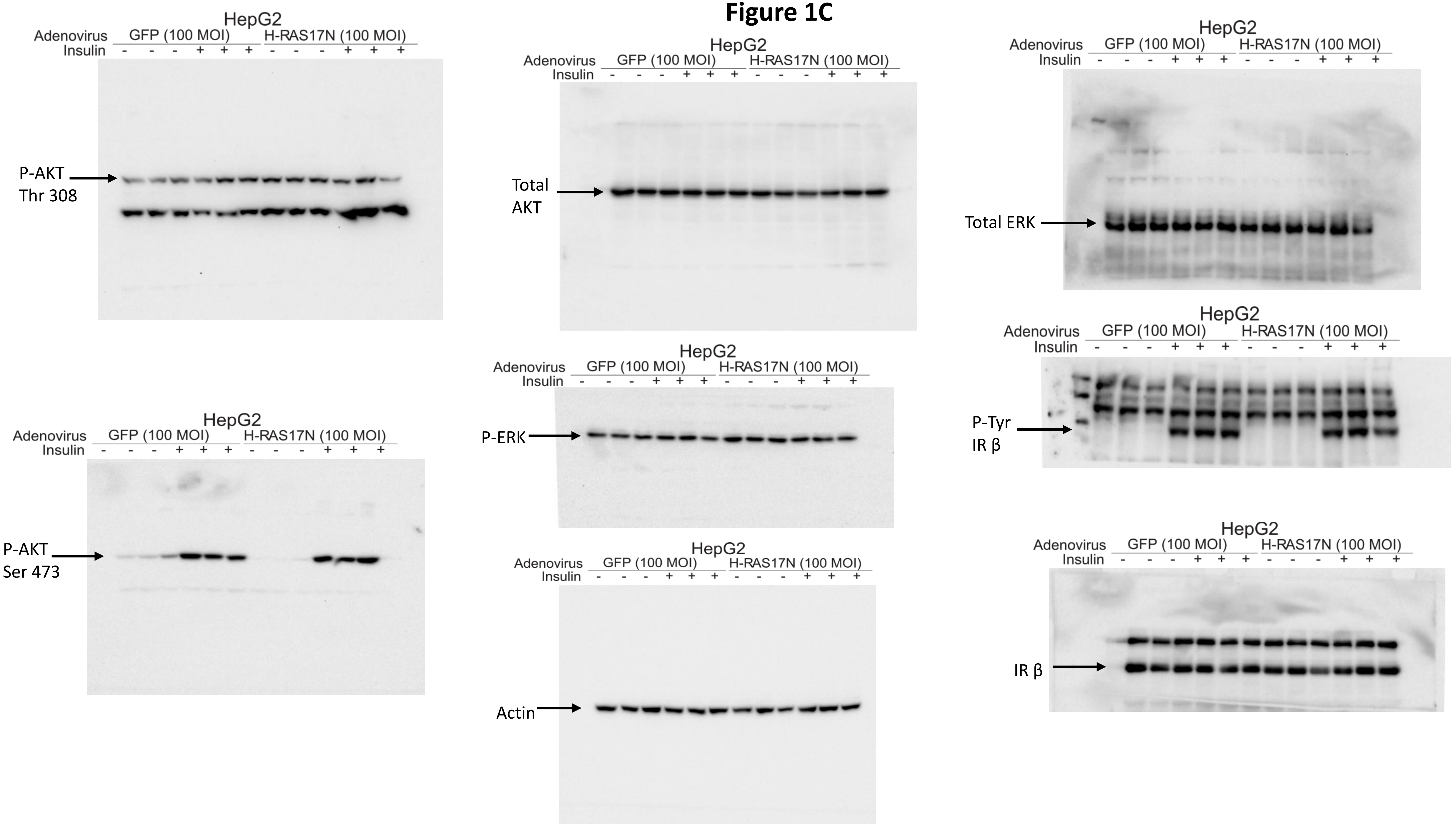
HepG2
Adenovirus GFP (100 MOI) H-RAS17N (100 MOI)
Insulin - - - + + + - - - + + +
P-AKT Thr 308
HepG2
Adenovirus GFP (100 MOI) H-RAS17N (100 MOI)
Insulin - - - + + + - - - + + +
P-AKT Ser 473
HepG2
Adenovirus GFP (100 MOI) H-RAS17N (100 MOI)
Insulin - - - + + + - - - + + +
Total AKT
HepG2
Adenovirus GFP (100 MOI) H-RAS17N (100 MOI)
Insulin - - - + + + - - - + + +
P-ERK
HepG2
Adenovirus GFP (100 MOI) H-RAS17N (100 MOI)
Insulin - - - + + + - - - + + +
Actin
HepG2
Adenovirus GFP (100 MOI) H-RAS17N (100 MOI)
Insulin - - - + + + - - - + + +
Total ERK
HepG2
Adenovirus GFP (100 MOI) H-RAS17N (100 MOI)
Insulin - - - + + + - - - + + +
P-Tyr IR β
HepG2
Adenovirus GFP (100 MOI) H-RAS17N (100 MOI)
Insulin - - - + + + - - - + + +
IR β

Figure 2A

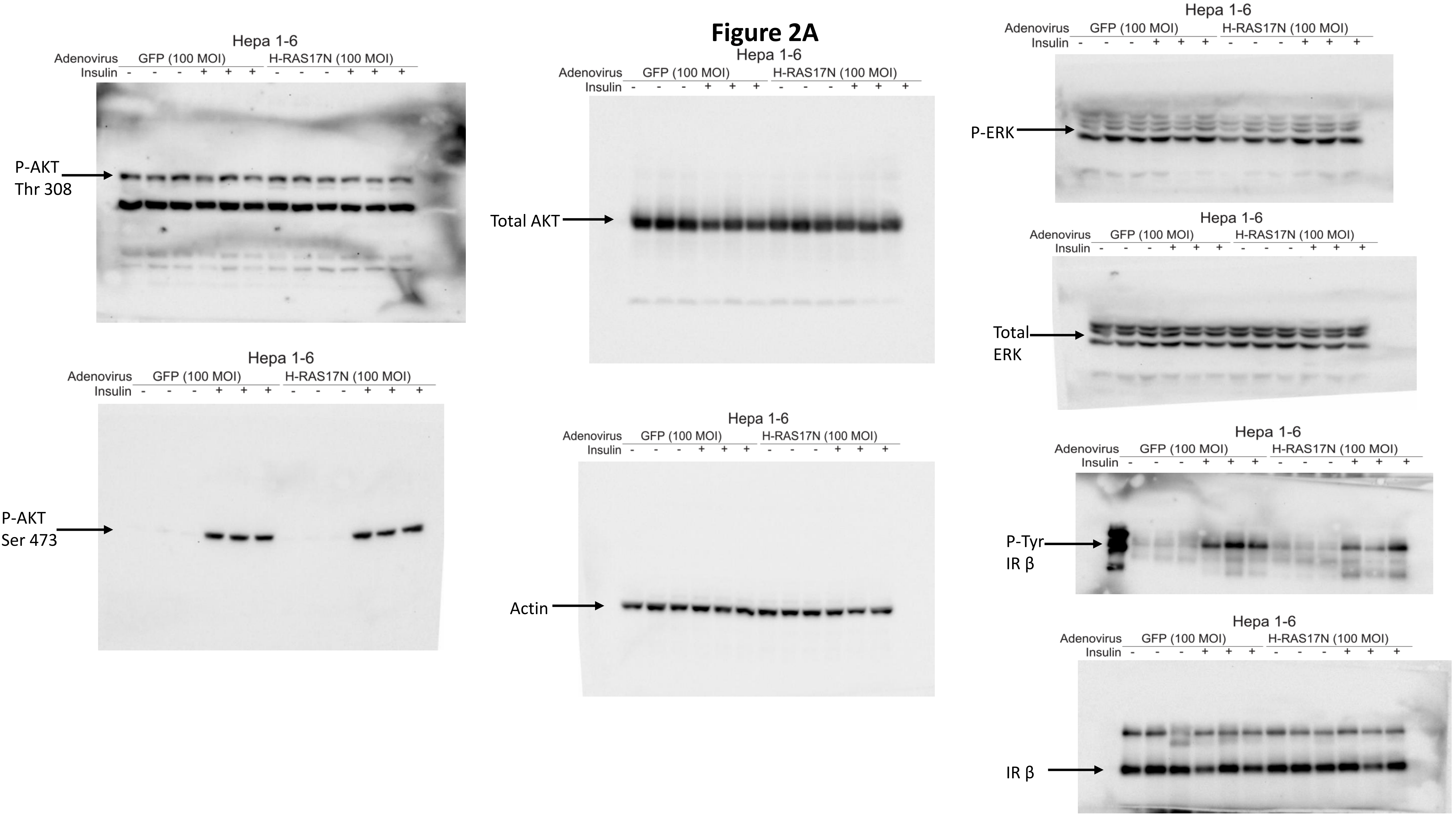
Hepa 1-6
Adenovirus GFP (100 MOI) H-RAS17N (100 MOI)
Insulin - - - + + + - - - + + +
P-AKT Thr 308
P-AKT Ser 473
Total AKT
Actin
P-ERK
Total ERK
P-Tyr IR β
IR β

Figure 2C

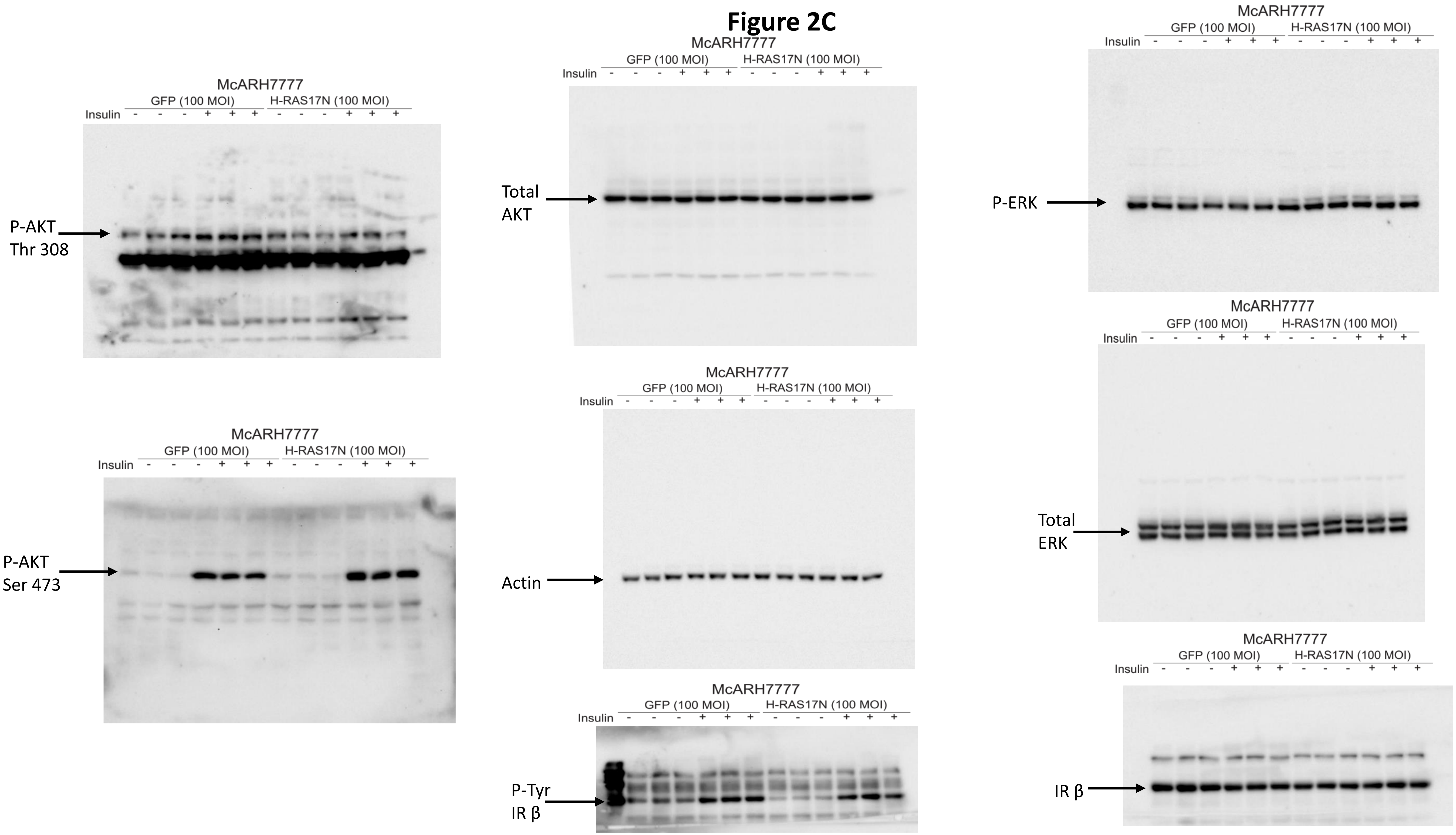
McARH7777
GFP (100 MOI)
H-RAS17N (100 MOI)
Insulin - - - + + + - - - + + +
P-AKT Thr 308
P-AKT Ser 473
Total AKT
Actin
P-Tyr IR β
P-ERK
Total ERK
IR β

Figure 3F

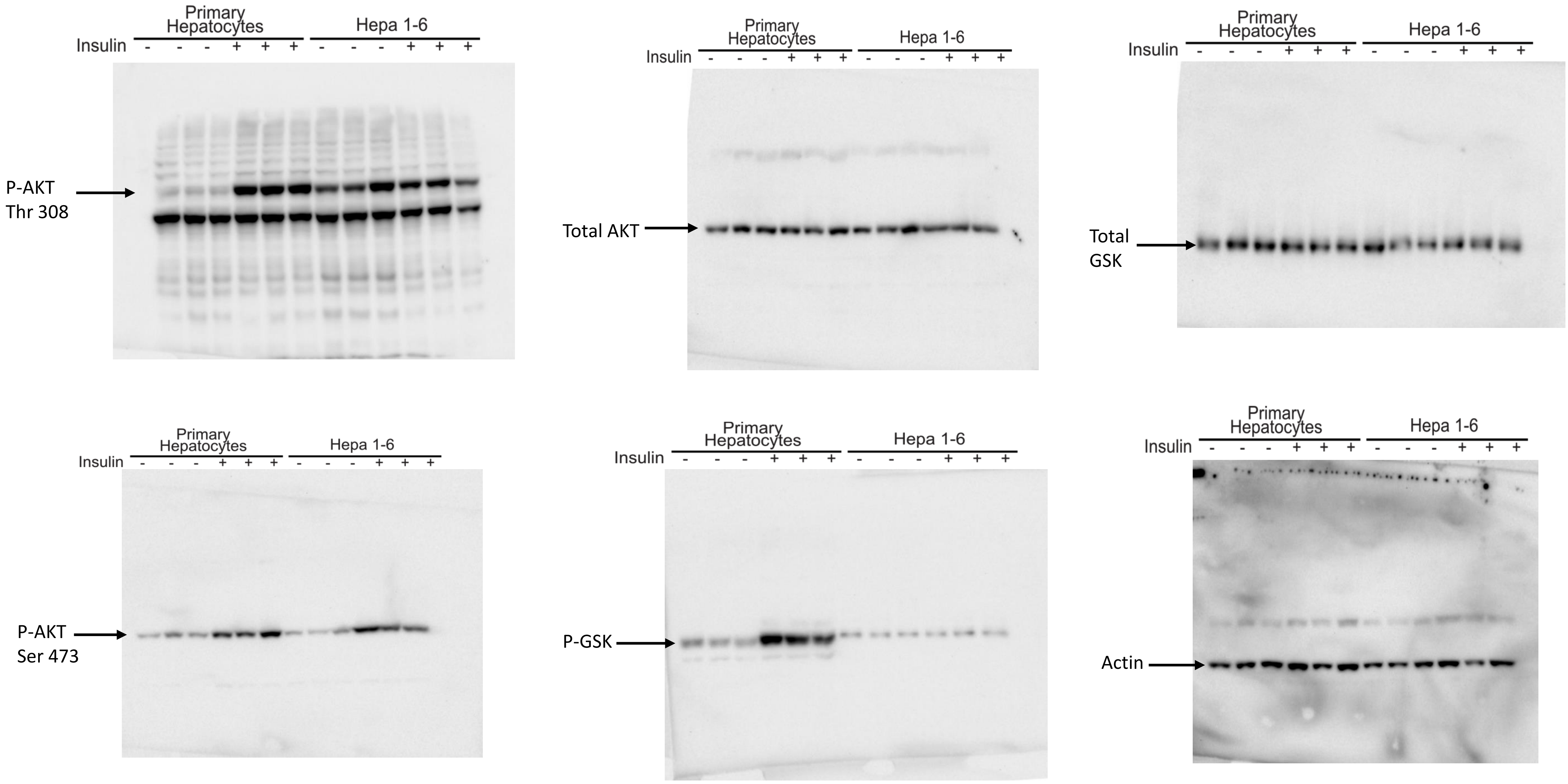

Primary Hepatocytes
Hepa 1-6
Insulin - - - + + + - - - + + +
P-AKT Thr 308
Total AKT
Total GSK
P-AKT Ser 473
P-GSK
Actin

Figure 4A

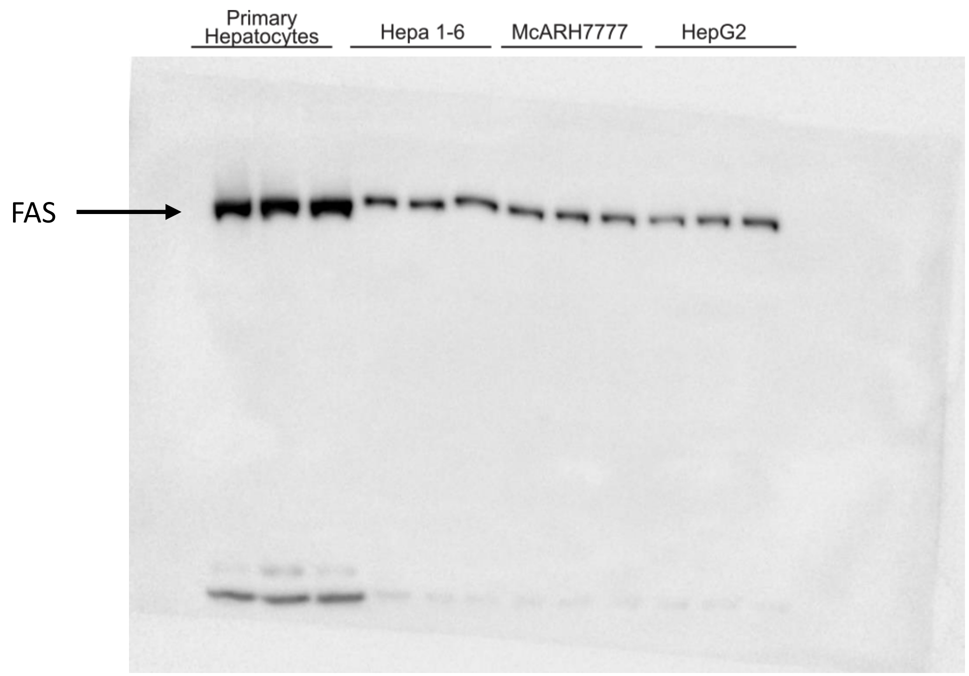

Primary Hepatocytes
Hepa 1-6
McARH7777
HepG2
FAS

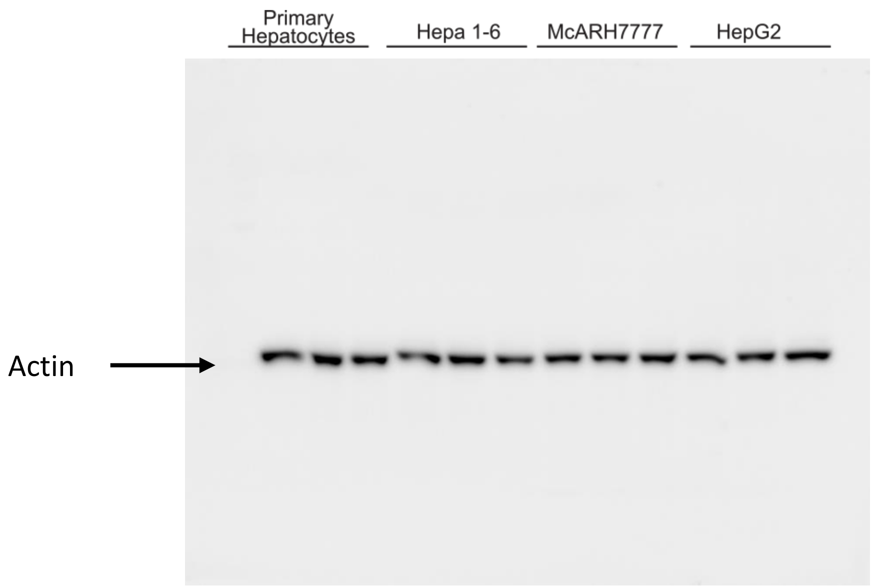

Primary Hepatocytes
Hepa 1-6
McARH7777
HepG2
Actin

Figure S2A

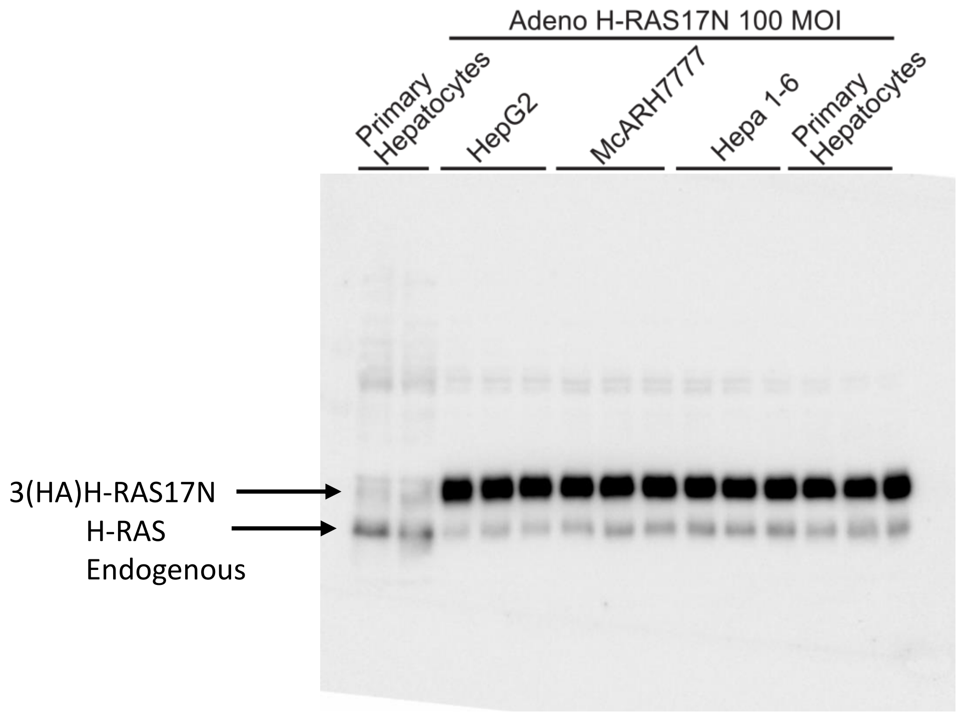

Adeno H-RAS17N 100 MOI
Primary Hepatocytes
HepG2
McARH7777
Hepa 1-6
Primary Hepatocytes
3(HA)H-RAS17N
H-RAS Endogenous

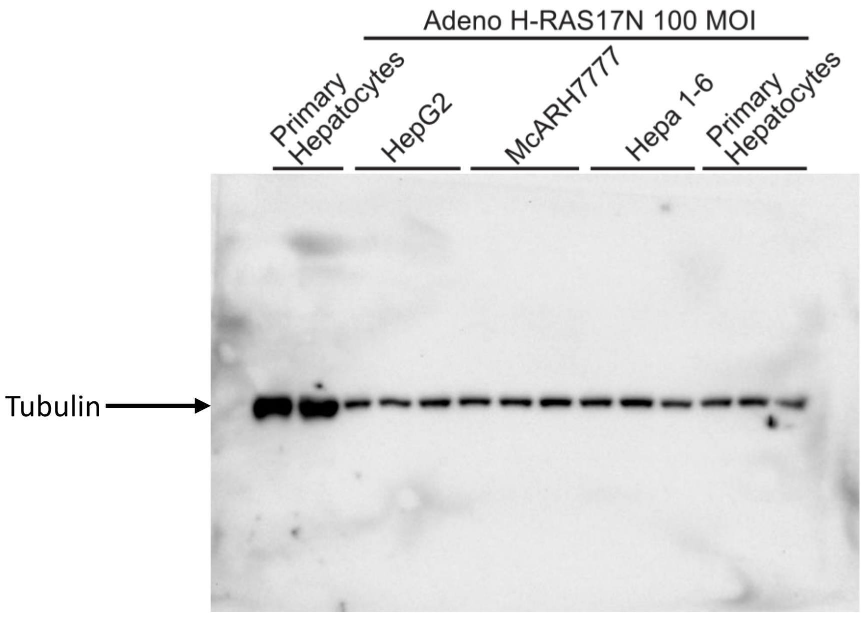

Adeno H-RAS17N 100 MOI
Primary Hepatocytes
HepG2
McARH7777
Hepa 1-6
Primary Hepatocytes
Tubulin

Figure S3A

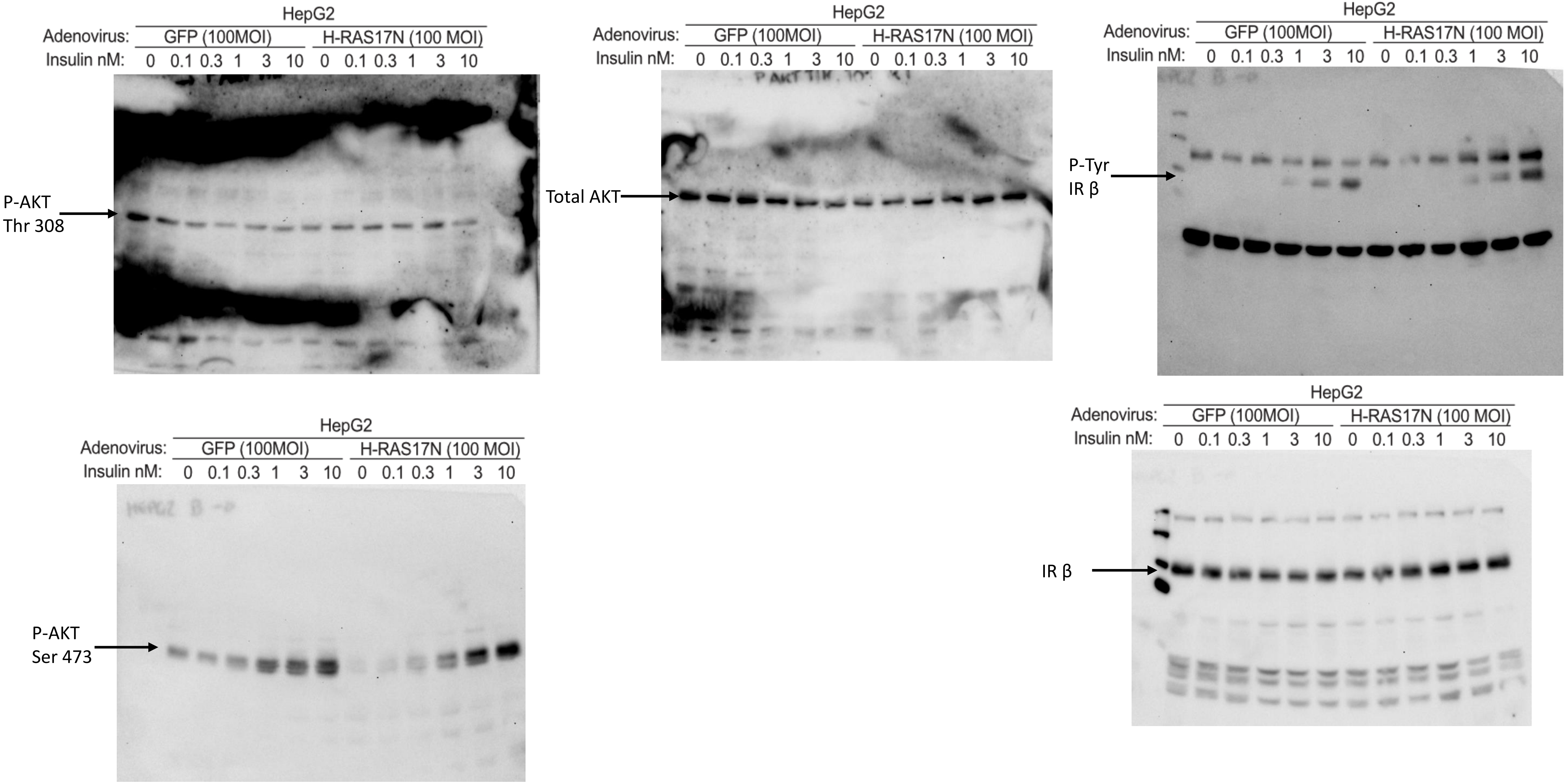
HepG2
Adenovirus: GFP (100MOI) H-RAS17N (100 MOI)
Insulin nM: 0 0.1 0.3 1 3 10 0 0.1 0.3 1 3 10
P-AKT
Thr 308
HepG2
Adenovirus: GFP (100MOI) H-RAS17N (100 MOI)
Insulin nM: 0 0.1 0.3 1 3 10 0 0.1 0.3 1 3 10
Total AKT
HepG2
Adenovirus: GFP (100MOI) H-RAS17N (100 MOI)
Insulin nM: 0 0.1 0.3 1 3 10 0 0.1 0.3 1 3 10
P-Tyr
IR β
HepG2
Adenovirus: GFP (100MOI) H-RAS17N (100 MOI)
Insulin nM: 0 0.1 0.3 1 3 10 0 0.1 0.3 1 3 10
P-AKT
Ser 473
HepG2
Adenovirus: GFP (100MOI) H-RAS17N (100 MOI)
Insulin nM: 0 0.1 0.3 1 3 10 0 0.1 0.3 1 3 10
IR β

Figure S3B

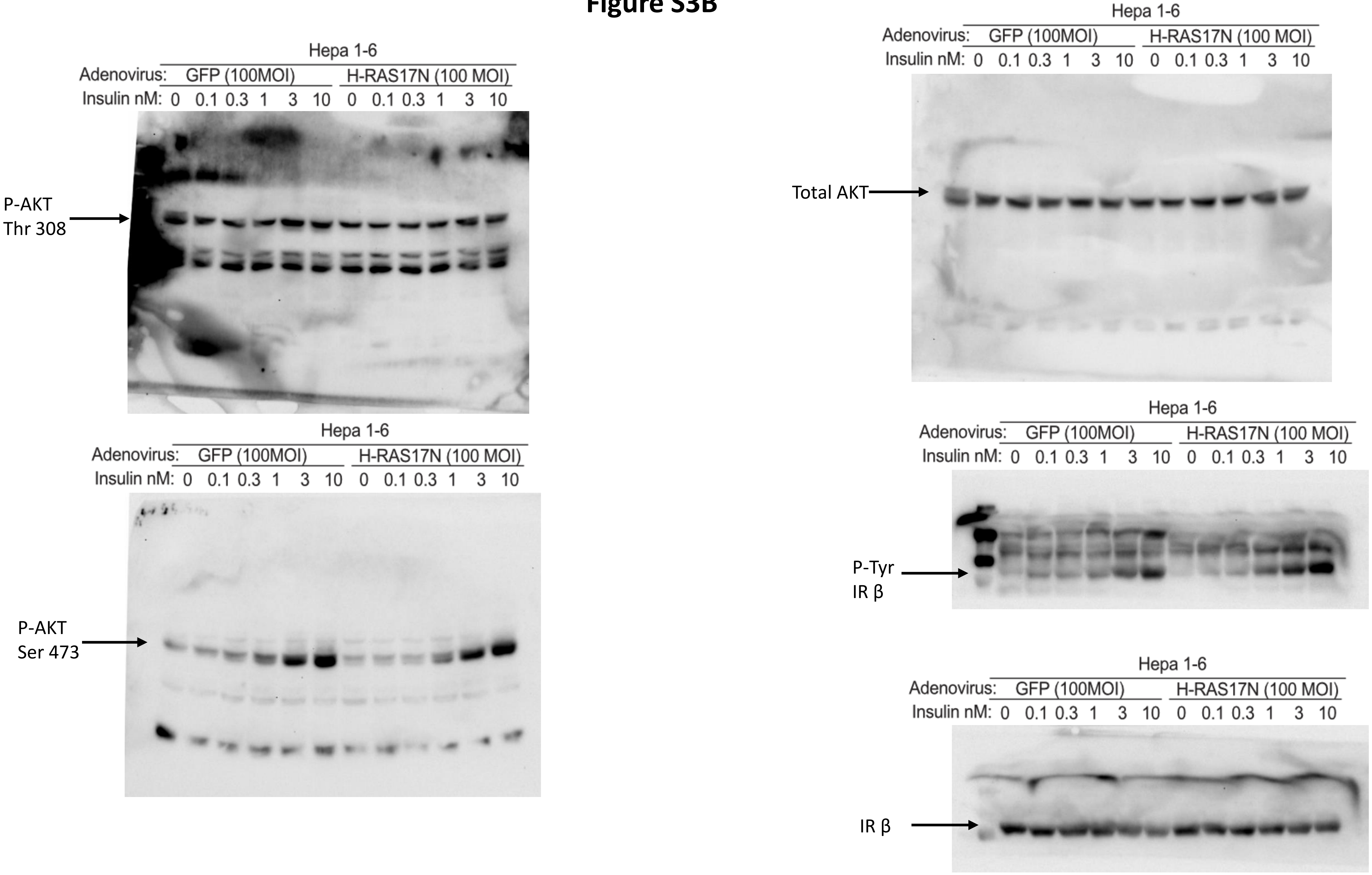

Hepa 1-6
Adenovirus: GFP (100MOI) H-RAS17N (100 MOI)
Insulin nM: 0 0.1 0.3 1 3 10 0 0.1 0.3 1 3 10
P-AKT Thr 308
Hepa 1-6
Adenovirus: GFP (100MOI) H-RAS17N (100 MOI)
Insulin nM: 0 0.1 0.3 1 3 10 0 0.1 0.3 1 3 10
P-AKT Ser 473
Hepa 1-6
Adenovirus: GFP (100MOI) H-RAS17N (100 MOI)
Insulin nM: 0 0.1 0.3 1 3 10 0 0.1 0.3 1 3 10
Total AKT
Hepa 1-6
Adenovirus: GFP (100MOI) H-RAS17N (100 MOI)
Insulin nM: 0 0.1 0.3 1 3 10 0 0.1 0.3 1 3 10
P-Tyr IR β
Hepa 1-6
Adenovirus: GFP (100MOI) H-RAS17N (100 MOI)
Insulin nM: 0 0.1 0.3 1 3 10 0 0.1 0.3 1 3 10
IR β

Figure S3C

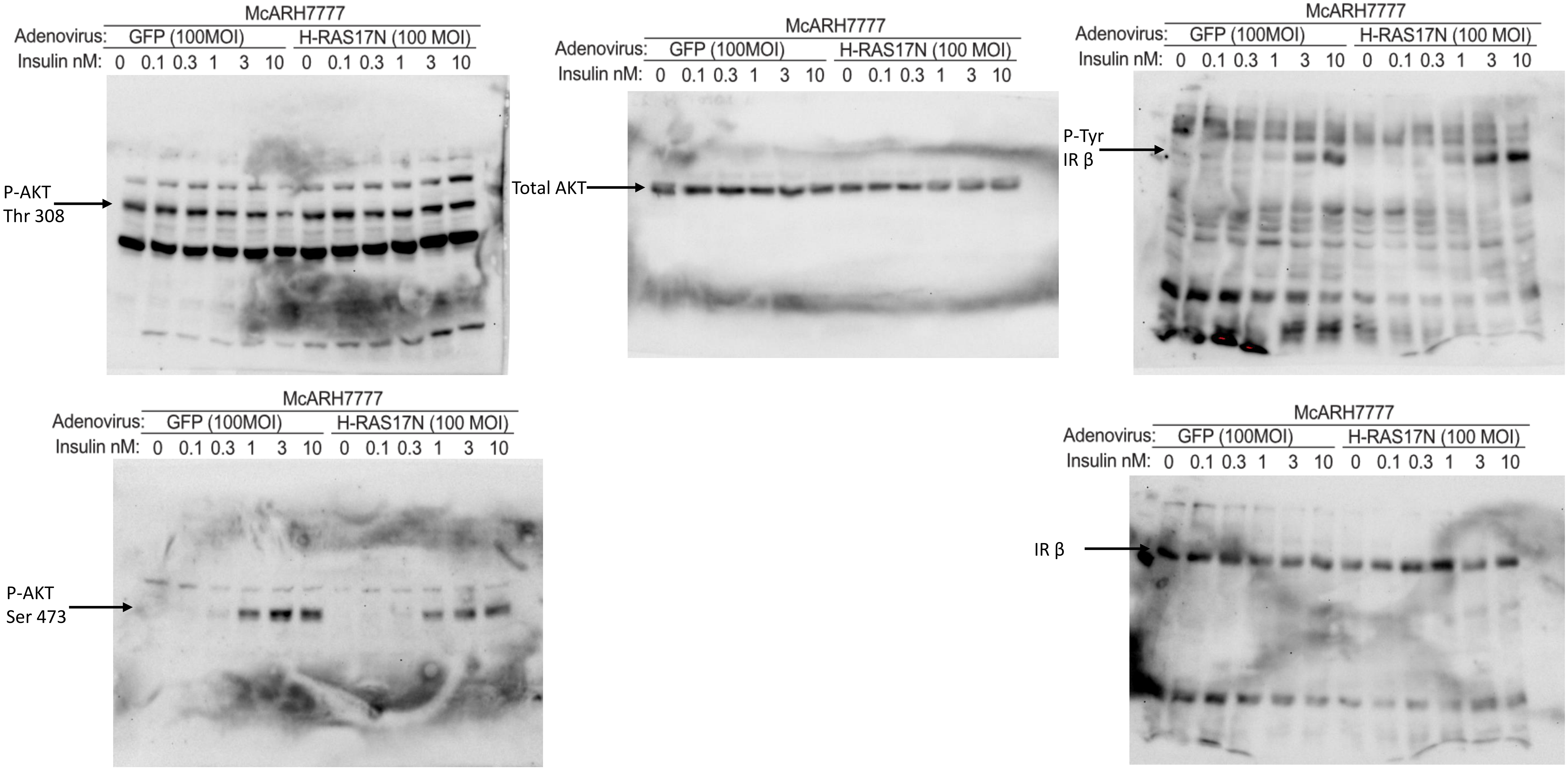
McARH7777
Adenovirus: GFP (100MOI) H-RAS17N (100 MOI)
Insulin nM: 0 0.1 0.3 1 3 10 0 0.1 0.3 1 3 10
P-AKT Thr 308
McARH7777
Adenovirus: GFP (100MOI) H-RAS17N (100 MOI)
Insulin nM: 0 0.1 0.3 1 3 10 0 0.1 0.3 1 3 10
Total AKT
McARH7777
Adenovirus: GFP (100MOI) H-RAS17N (100 MOI)
Insulin nM: 0 0.1 0.3 1 3 10 0 0.1 0.3 1 3 10
P-Tyr IR β
McARH7777
Adenovirus: GFP (100MOI) H-RAS17N (100 MOI)
Insulin nM: 0 0.1 0.3 1 3 10 0 0.1 0.3 1 3 10
P-AKT Ser 473
McARH7777
Adenovirus: GFP (100MOI) H-RAS17N (100 MOI)
Insulin nM: 0 0.1 0.3 1 3 10 0 0.1 0.3 1 3 10
IR β

Figure S4A

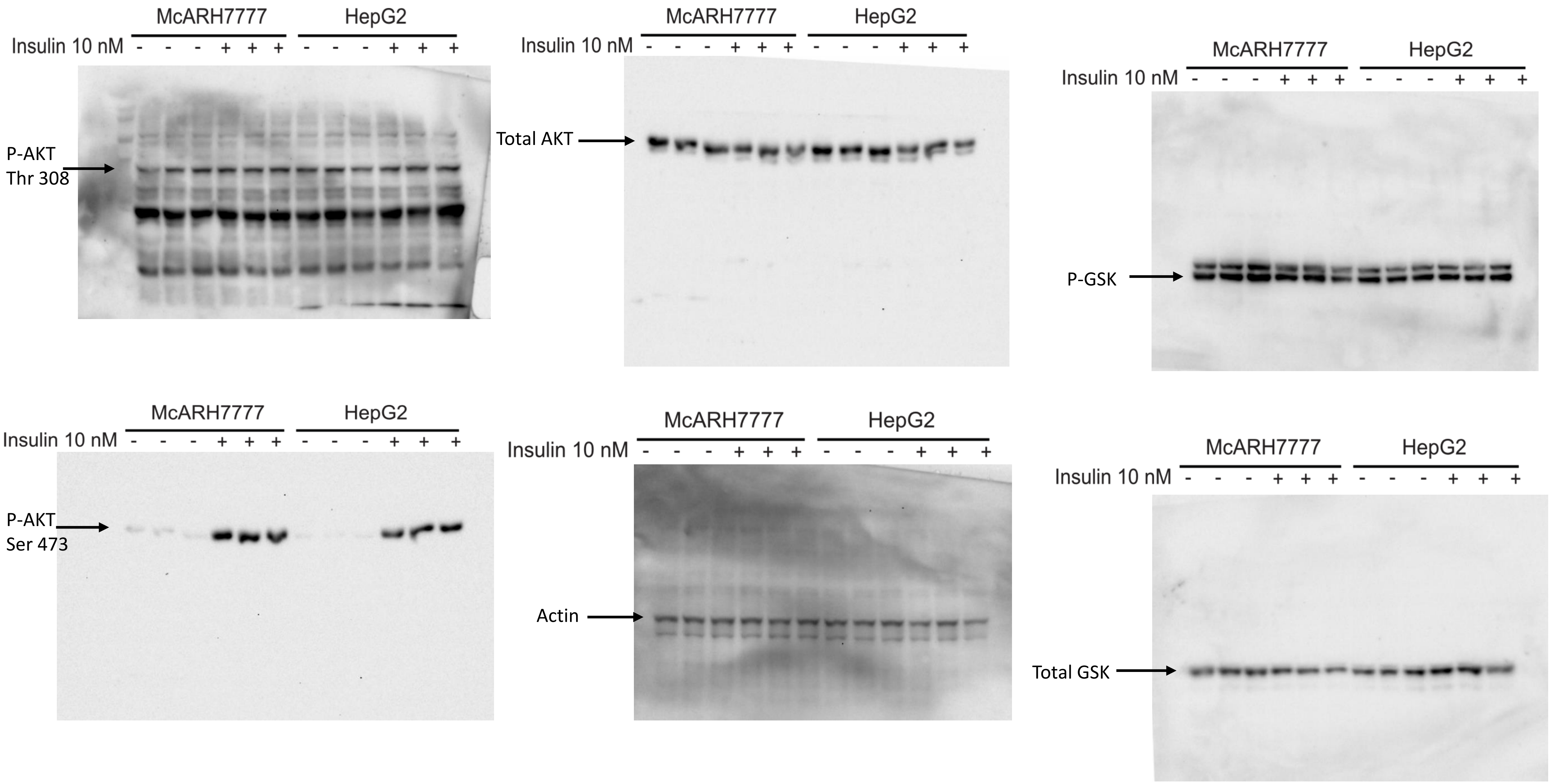
McARH7777
HepG2
Insulin 10 nM - - - + + + - - - + + +
P-AKT Thr 308
Total AKT
P-GSK
P-AKT Ser 473
Actin
Total GSK

Figure S4C

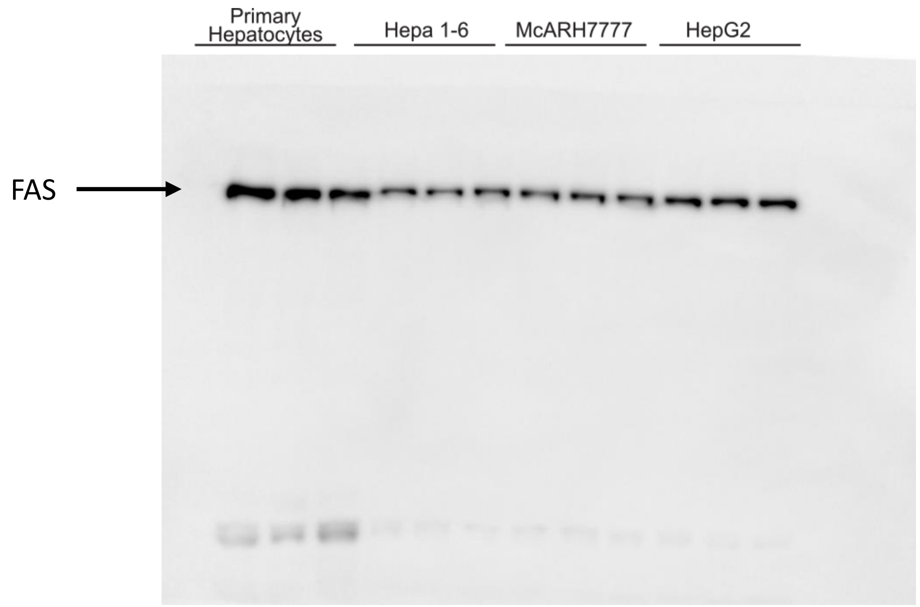
Primary Hepatocytes
Hepa 1-6
McARH7777
HepG2
FAS

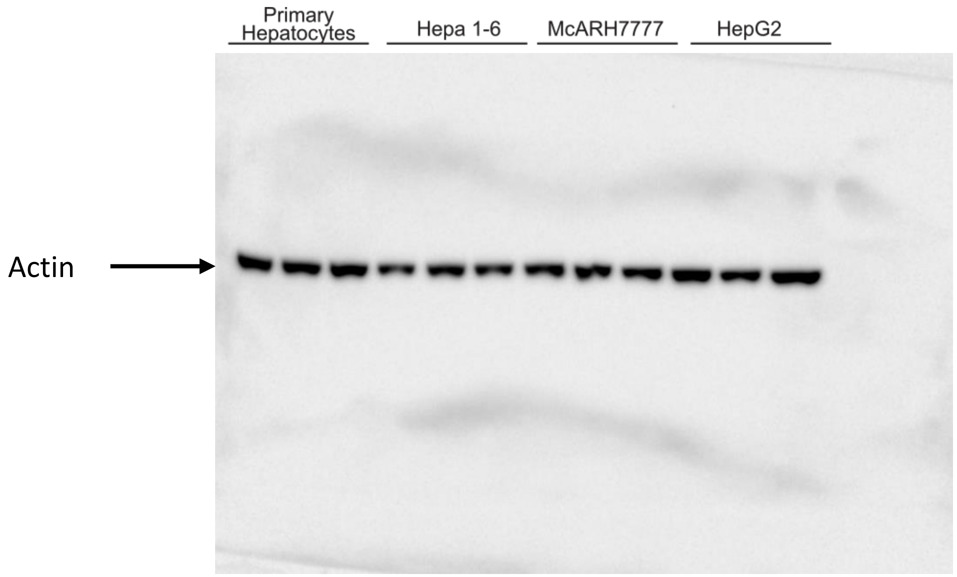
Primary Hepatocytes
Hepa 1-6
McARH7777
HepG2
Actin

Figure S4D

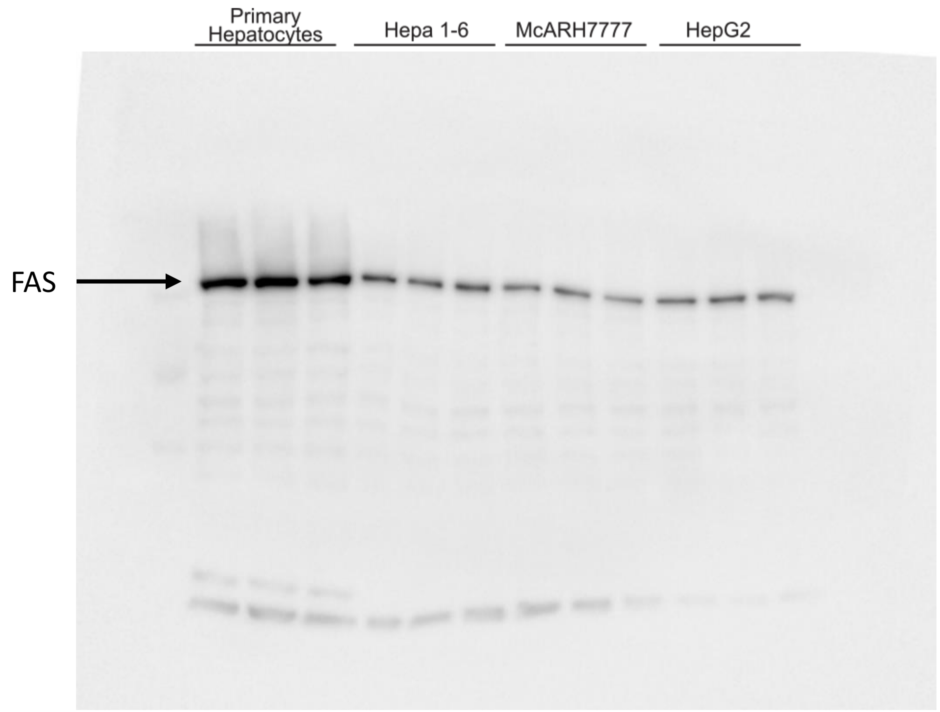
Primary Hepatocytes
Hepa 1-6
McARH7777
HepG2
FAS

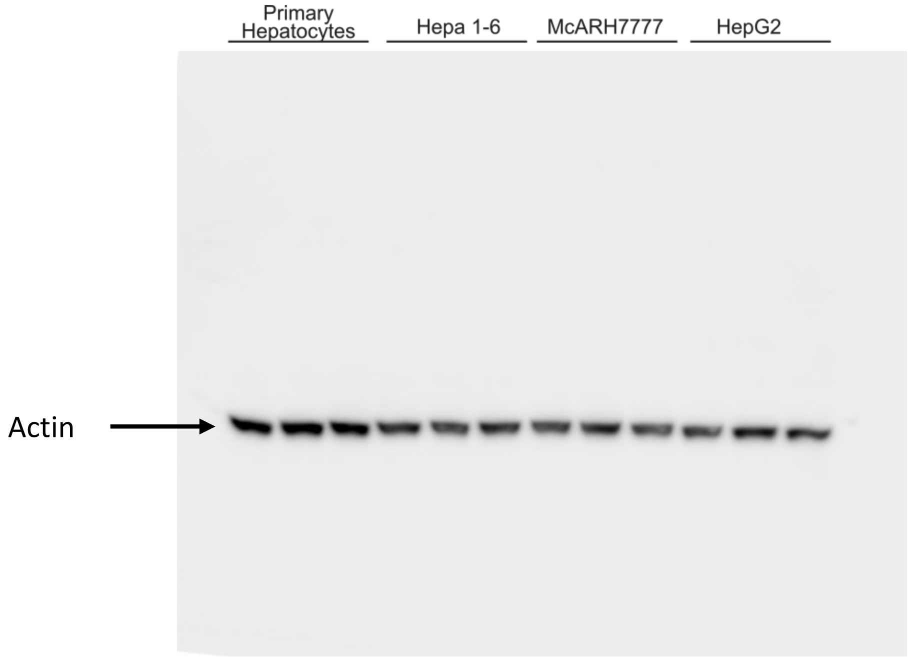
Primary Hepatocytes
Hepa 1-6
McARH7777
HepG2
Actin
